# Systemically functional characterization of regiospecific flavonoid O-methyltransferases from *Glycine max*

**DOI:** 10.1016/j.synbio.2024.03.009

**Published:** 2024-03-15

**Authors:** Bingtong Feng, Yuguo Jiang, Xiaodong Li, Yan Wang, Ziyu Ren, Jian Lu, Xing Yan, Zhihua Zhou, Pingping Wang

**Affiliations:** a Life Science and Technology College, Guangxi University, Nanning, Guangxi, 530004, China; b CAS-Key Laboratory of Synthetic Biology, CAS Center for Excellence in Molecular Plant Sciences, Chinese Academy of Sciences, Shanghai, 200032, China; c University of Chinese Academy of Sciences, Beijing, 100049, China; d The College of Life Sciences, Shanghai Normal University, Shanghai, 200234, China

**Keywords:** Flavonoids, O-methyltransferases, *Glycine max*, Functional characterization

## Abstract

Plants produce diverse flavonoids for defense and stress resistance, most of which have health benefits and are widely used as food additives and medicines. Methylation of the free hydroxyl groups of flavonoids, catalyzed by *S*-adenosyl-l-methionine-dependent O-methyltransferases (OMTs), significantly affects their physicochemical properties and bioactivities. Soybeans (*Glycine max*) contain a rich pool of O-methylated flavonoids. However, the OMTs responsible for flavonoid methylation in *G.* max remain largely unknown. We screened the *G.* max genome and obtained 22 putative OMT-encoding genes that share a broad spectrum of amino acid identities (25–96%); among them, 19 OMTs were successfully cloned and heterologously expressed in *Escherichia coli*. We used the flavonoids containing the free 3, 5, 7, 8, 3′, 4′ hydroxyl group, such as flavones (luteolin and 7, 8-dihydroxyflavone), flavonols (kaempferol and quercetin), flavanones (naringenin and eriodictyol), isoflavonoids (daidzein and glycetein), and caffeic acid as substrates, and 15 OMTs were proven to catalyze at least one substrate. The methylation activities of these GmOMTs covered the 3, 7, 8, 3′, 4′- hydroxyl of flavonoids and 7, 4′- hydroxyl of isoflavonoids. The systematic characterization of *G*. max flavonoid OMTs provides insights into the biosynthesis of methylated flavonoids in soybeans and OMT bioparts for the production of methylated flavonoids via synthetic biology.

## 1 Introduction

Flavonoids are a large group of plant secondary metabolites found in many plants, and the estimated number of members is greater than 6000 [1,2]. Flavonoids protect plants from UV irradiation and microbial infection [3,4]. Some flavonoids, for example, rhamnetin [5,6], genkwanin [7,8], and kaempferol [9,10], have anti-inflammatory, anti-bacterial, anti-melanogenesis, anti-tumor and anti-cholesterol activities, which have potential in medicines and healthcare products [11,12]. Many flavonoids exist in plants in a methylated form, and methylation of the free hydroxyl of flavonoids significantly changes their physiochemical properties and bioactivities by altering their reactivity, solubility, and interaction with other molecules [[13], [14], [15]]. Approximately 1600 O-methoxylated flavonoid derivatives have been identified in plants [16], and plants from Leguminosae family, especially *Glycine max*, produce various methoxylated flavonoids, such as glycitin, 4′-methoxygenistein, isorhamnetin, afromosin, and formononetin [[17], [18], [19]].

O-Methyltransferase (OMT) mediates the transfer of a methyl group from *S*-adenosyl-l-methionine (SAM) to the hydroxyl group of natural products, producing the methylated product and by-product *S*-adenosyl-l-homocysteine (SAH) [20,21]. Plant OMTs are divided into three different families (type I to III) based on protein sequence and structure [22]. Type I OMTs typically have a relative molecular mass of 38–45 KD and their activity is independent of Mg2+, most of the plant flavonoids OMTs belong to this family [23]. Type II OMTs have a smaller relative molecular mass of 22–27 KD and their activity is dependent on Mg2+, they are mainly involved in lignin biosynthesis and recent studies have showed that they could also catalyze the methylation of flavonoids [23]. Plant OMTs are a superfamily of genes with many candidate genes predicted from the genome; for example, 58 potential OMTs have been predicted in citrus [24], 47 in *Vitis vinifera* [25], 26 in *Populus trichocarpa* [26], 82 in *Gossypium hirsutum*, 55 in *Gossypium arboreum*, and 55 in *Gossypium Raimondii* [27]; however, most have not been functionally characterized. Thirty-eight OMTs from different plants have been characterized to catalyze the O-methylation of flavonoids, typically in the 3, 7, 8, 3′, 4′- hydroxyl of flavonoids and 7, 4′- hydroxyl of isoflavonoids. Although *G*. max produces many methylated flavonoids, only one OMT (SOMT2) that catalyzes the 4′-OH methylation of daidzein, genistein, and naringenin has been reported [28].

The utilization of SAM as a common methyl donor and flavonoids as methyl acceptors suggests that conserved motifs exist for SAM binding or substrate recognition. After comparing five characterized MTs from different species, Bugos et al. suggested that five conserved regions contribute to SAM binding [29]. Kagan and Clarke also found three conserved domains in 84 MTs [30]. However, these early attempts employed only a limited number of MTs for motif prediction, and none of the MTs were characterized as flavonoid OMTs. In 1998, Joshi and Chiang proposed three SAM-binding domains (motifs A, B, and C) and four putative substrate-recognition domains (motifs I, J, K, and L) based on an analysis of 56 plant SAM-dependent MT sequences [31]. The functional characterization of additional FOMTs would improve the understanding of this unique conserved information of FOMTs, facilitating the annotation of putative FOMTs in plant genomes and the understanding of structure-function relationships between protein sequences and their substrate specificities and, thus, improving the prediction of unknown OMTs.

**Fig. 1:**
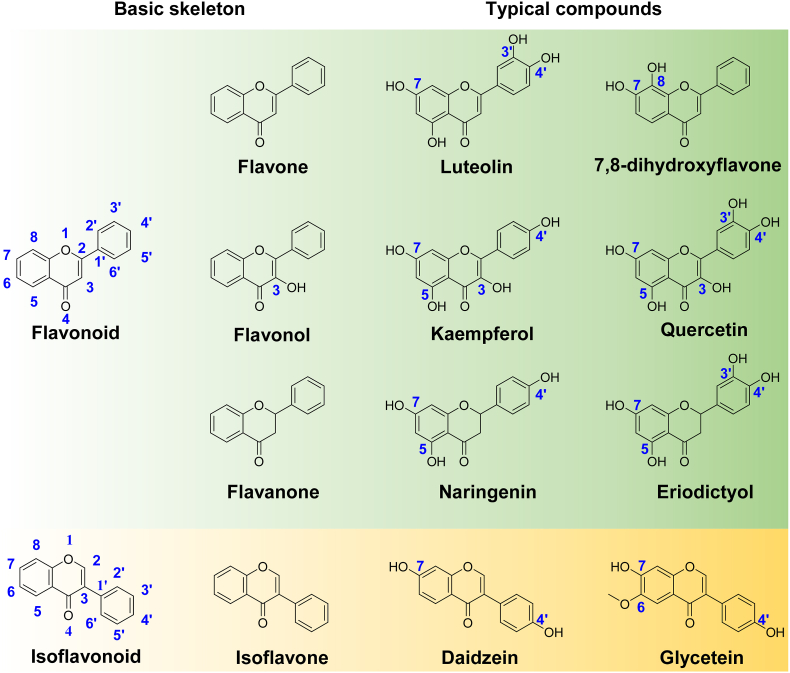
Structure of flavonoid substrates used for GmOMTs' activities assay.

In this study, we aimed to systematically characterize flavonoid O-methyltransferases from *G.* max at the genome scale. First, we attempted to discover all potential OMT candidates in *G.* max by screening the whole genome using the reported FOMT as a query. Candidates were cloned from *G.* max and heterologously expressed in *Escherichia coli*. A series of general flavonoid compounds containing free 3-, 5-, 7-, 8-, 3′, 4’ -hydroxyl groups were used as substrates to test the enzymatic activities of the candidates (Fig. 1). On the basis of phylogenetic analysis and sequence alignment, we proposed seven novel conserved motifs with improved accuracy for FOMTs, which could facilitate the discovery and functional prediction of unknown plant OMTs. The systematic functional characterization of a series of FOMTs helps explain the biosynthesis and regulation of methylated flavonoids in *G*. max and provides OMT bioparts with diverse substrates and regiospecificity for the production of methylated flavonoids in synthetic biology.

## 2 Materials and methods

### 2.1 Materials and reagents

*Glycine* max (soybean) was grown in greenhouse of Center for Excellence in Molecular Plant Science, Chinese Academy of Sciences; *E. coli* JM109 was used for gene cloning and *E. coli* BL21 (DE3) was used for heterologous expression of GmOMTs. Flavonoid standards were purchased from Nantong Feiyu Biological Technology (Nantong, China).

### 2.2 Cloning of OMTs from *Glycine max*

Soybean leaves were frozen in liquid nitrogen, ground in a mortar with pestle to fine powder. Total RNA was extracted using RNAprep Pure Plant Kit (Polysaccharides& Polyphenolics-rich) (TIANGEN, China), cDNA was prepared using PrimerScript™ RT reagent Kit with gDNA Eraser (TAKARA, Japan). The soybean OMT candidates were identified by screening the entire genome of *Glycine* max downloaded from SoyCyc 8.0 (https://www.plantcyc.org/databases/soycyc/8.0) using SOMT2 (GeneBank accession number: TC178411) as a query for BLAST searches. Candidate genes which encoding more than 350 amino acids and protein sequence identities >40% to SOMT2 were selected for cloning. Primers were designed according to the candidates’ sequences and ordered from Sangon-Biotech (Shanghai, China). Polymerase chain reactions (PCR) using soybean cDNA as template were performed with I-5™ High-Fidelity DNA Polymerase (TSINGKE Biological Technology, China). The PCR products were then subcloned into pMD18-T vector (TAKARA, Japan) and subjected to gene sequencing. The primers used for OMTs cloning were list in Table S1, the sequences of GmOMT1-22 were deposited in Table S2 (deposited in http://npbiosys.scbit.org/under accession No. OENC18–OENC36).

### 2.3 Phylogenetic and motif analysis of OMTs

The alignment of protein sequences was performed using MEGA 11 ClustalW with gap open penalty = 10 and gap extension penalty = 0.2 [32]. The phylogenetic tree was constructed by the program MEGA 11, using the neighbour-joining method with a 1000-replicate bootstrap search [32]. Motif analysis of plant flavonoid OMTs was performed using WebLogo [33].

### 2.4 Heterologous expression and function assay of *G*. max OMTs

The coding sequences of the 19 candidate GmOMTs was inserted into the pGEX4T-1 vector, respectively, the GmOMTs was expressed as N-terminal GST-fusion form. Recombinant pGEX4T-1 vector was transformed into *E. coli* BL21 (DE3). Inoculate the overnight cultured recombinant *E. coli* solution into a medium containing 50 ml LB and ampicillin, and culture at 37 °C, 150 rpm until OD_600_ = 0.4–0.6. IPTG with the final concentration of 200 μM was added and cultured at 18 °C, 110 rpm for about 18 h to complete the induced expression of the protein. The recombinant *E. coli* was collected by centrifugation at 4 °C and resuspended with 6 ml of 100 mM Tris-HCl pH = 8.0. The recombinant *E. coli* liquid was lysed by ultrasonic, and the supernatant was collected and used as crude enzyme for the following assays. The reaction was performed in a 200 μl volume containing 100 mM Tris-HCl buffer (pH 8.0), 500 μM SAM, 100 μM acceptor substrate, and 50 μl crude enzyme liquid in 30 °C water bath overnight and was terminated by adding 200 μl of ethyl acetate. The product was extracted into the organic phase, which was then evaporated. The residue was dissolved in methanol for subsequent assays.

### 2.5 High Performance Liquid Chromatography analysis

A Shimadzu LC-20A prominence system was used for the High Performance Liquid Chromatography (HPLC) analysis. Chromatographic separations were carried out at 35 °C on a Shodex C18-120-5 4E column (5 mm, 4.6 mm 3250 mm). The gradient elution system consisted of water (A) and acetonitrile (B). The HPLC program for the extracted compounds from the in vitro reactions by incubating GmOMTs with flavonoids as substrates was as follows: 0 min (22.5% B), 0–55 min (62.5% B), and 55–60 min (22.5% B). The flow rate was kept at 0.8 ml/min.

### 2.6 Mass spectrometry and nuclear magnetic resonance spectrometry

Mass spectrometry analysis was conducted on a Q-TOF 6520A mass spectrometer (Agilent Technologies, Germany) equipped with an ESI interface. The mass scan range was set from *m*/*z* 100 to 3000 in positive mode. The ion source parameters were: drying gas (N2) flow rate 9 L/min and temperature 345 °C; nebulizer pressure 38 psig; capillary voltage 3400V; skimmer 64V; OCT RF Vpp 750V and fragment 160V. The raw *m*/*z* data was processed with MassHunter Qualitative Analysis software (Agilent Technologies, version B.06). Nuclear magnetic resonance spectrometry (NMR) experiments were performed in (CD3)2SO for flavonoids on a Bruker Avance III 400 (for 1H NMR) (Bruker, Billerica, MA, USA). All spectra were referenced to residual protic resonance of solvent at 2.51 ppm.

## 3 Results and discussion

### 3.1 Annotation and cloning of GmOMT candidates from *Glycine max*

To discover potential OMTs in *G.* max at the genome-scale level, we used a previously reported *G.* max O-methyltransferase, SOMT2 [28], as a query to search for putative homologs in *G.* max protein database. Two criteria, a full open reading frame of more than 350 amino acids in length and protein sequence identity to SOMT2 higher than 40%, were used to identify putative *G.* max OMT homologs. In total, 22 OMT candidates (designated GmOMT1-22) were obtained (Table S2). They were between 26.3% and 96.7% identical at the amino acid level and contained all three conserved motifs in plant SAM-dependent methyltransferases [30]. Primers were designed according to the candidate GmOMTs’ sequences, and 19 of the 22 full-length GmOMTs were cloned and sequenced using *G*. max cDNA as a template. The 19 OMTs were GmOMT1-12 and 14–20.

### 3.2 Phylogenetic analysis of GmOMTs and characterized plant OMTs

**Fig. 2:**
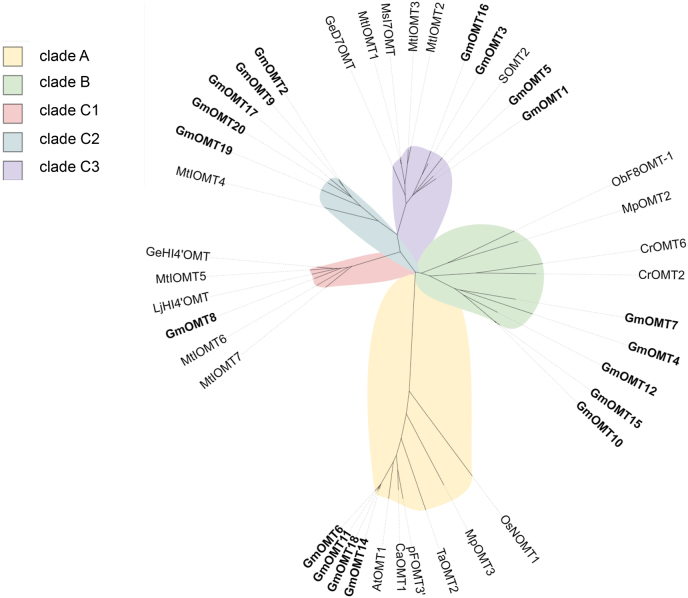
**Phylogenetic analysis of GmOMTs and previously characterized plant flavonoid OMTs**. Nineteen GmOMTs cloned in this study (marked in bold), and 22 characterized plant OMTs were used to generate the phylogenetic tree. The clade and subclade of the phylogenetic tree are marked with different colors. GenBank accession numbers of characterized plant OMTs sequences are listed in Table S3.

In the further analysis of these GmOMT candidates, a phylogenetic analysis of the other 22 characterized plant FOMTs (Table S3) from different plants was conducted, in which three main clades were generated (Fig. 2). OMTs from different clades shared less than 40% amino acid sequence identity.

GmOMT6, 11, 14, 18, shared higher than 95% protein identity were classified into clade A with a series of flavonoids 3′-OMTs (e.g., AtOMT2 from *Arabidopsis thaliana*, CaOMT1, and PFOMT3′ from *Chrysosplenium americanum*, TaOMT2 from *Triticum aestivum* L. and MpOMT3 from *Mentha x piperita*) (Fig. 2). These OMTs were greater than 50% identical, and a 4′-OMT (i.e., OsNOMT1 from *Oryza sativa Japonica Group*) was less than 41% identical to other OMTs within this clade. A comparison of the OMTs in clade A and other clades demonstrated obvious differences in protein sequences, indicating that clade A has a distant evolutionary relationship with the other clades in the phylogenetic analysis (Fig. 2).

GmOMT4, 7, 10, 12, 15 of clade B shared 70–84% protein identity with each other and 42–55% protein identity with other OMTs within this clade (e.g., MpOMT2 from *Medicago truncatula*, ObF8OMT-1 from *Ocimum basilicum*, and CrOMT2 and CrOM6 from *Catharanthus roseus*) (Fig. 2).

Clade C was further divided into three subclades: C1, C2, and C3. (Fig. 2). Clade C1 contains GmOMT8 with five isoflavanones 4′-OMTs (i.e., MtIOMT5-7, GeHI4′OMT, and LjHI4′OMT from *Medicago truncatula*; *Glycyrrhiza echinate*; and *Lotus japonicus*) (Fig. 2). OMTs within clade C1 are 73–81% identical to each other at the protein level. MtIOMT4, a flavonoid 7-OH OMT and the only characterized OMT in clade C2 (Fig. 2), shared 62–66% protein sequence identity with GmOMT2, 9, 17, 19, 20. GmOMT1, 3, 5, and 16 shared 70–84% protein identity; they were classified into clade C3 with a series of isoflavonoid 7-OMTs (e.g., MtIOMT1-3 from *M. truncatula*, GeD7OMT from *Glycyrrhiza echinate*, MsI7OMT from *Medicago sativa*, and SOMT2 from *G. max*) (Fig. 2). OMTs within this clade were 60–84% identical at the protein level. The OMTs from *G*. max in clade C3 clustered in the same small clade, and the isoflavonoid 7-OMTs from other species clustered in another small clade (Fig. 2), indicating that the OMTs have a certain degree of species specificity in clade C3.

### 3.3 Substrate specificity of GmOMTs toward flavonoids

To test the substrate specificity of *G.* max OMTs, the 19 GmOMTs were heterologously expressed in *E. coli* and a series of flavonoid substrates containing 3, 5, 7, 8, 3′, 4’ –OH group, e.g. flavones (luteolin and 7, 8-dihydroxyflavone), flavonols (kaempferol and quercetin), flavanones (naringenin and eriodictyol), isoflavonoids (daidzein and glycetein), and caffeic acid were used as substrates for the enzyme assay (Fig. 1).

**Fig. 3:**
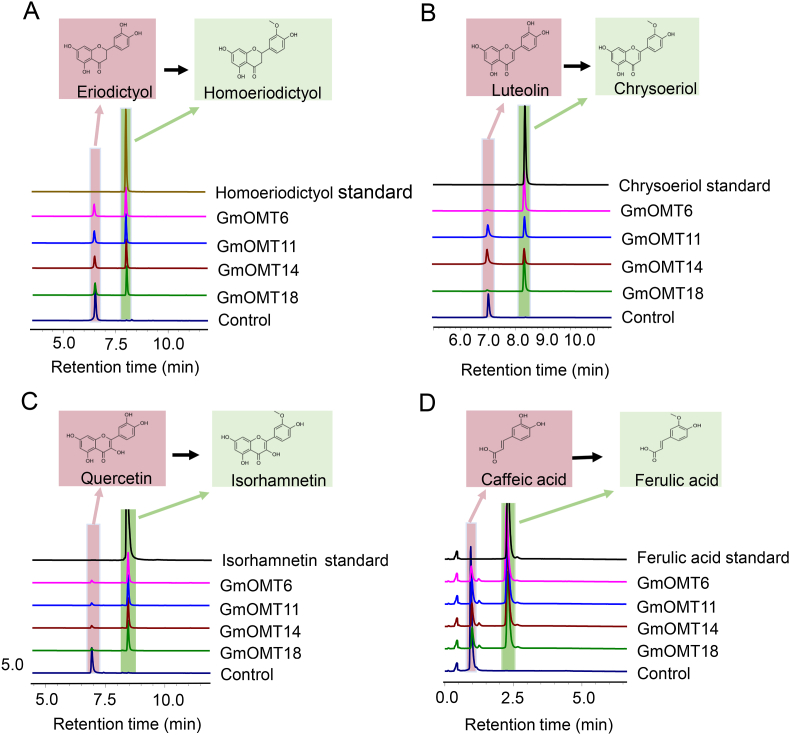
**Enzyme activities assay of GmOMT6, 11, 14, and 18 toward different flavonoid substrates**. HPLC analysis of the reaction products produced by incubating crude enzymes of GmOMT6, 11, 14, and 18 with eriodictyol (A), luteolin (B), quercetin (C), and caffeic acid (D) as the substrate, respectively. Crude enzyme made from *E. coli* strain with empty pGEX-4T-1vector was used as a negative control. Authentic standards homoeriodictyol, chrysoeriol, isorhamnetin, and ferulic acid were used for the verification of the corresponding methylated products produced by each OMT.

GmOMT6, 11, 14, and 18 were classified into clade A, and OMTs in this clade have been proven to catalyze the 3′-OH methylation of flavonoids (Fig. 2). The enzymatic assay results indicated that all four OMTs could catalyze the 3′-OH methylation of three flavonoids, eriodictyol, luteolin, and quercetin, producing the corresponding methylated products: homoeriodictyol (Fig. 3A), chrysoeriol (Fig. 3B) and isorhamnetin (Fig. 3C), respectively. These four GmOMTs can also methylate the 3′-OH of caffeic acid to yield ferulic acid (Fig. 3D). Results indicated that these four GmOMTs are flavonoids 3′-OH methyltransferases, consistent with other OMTs in this clade. Regarding enzyme catalytic efficiency, the four GmOMTs converted more than 80% of luteolin and quercetin into their methylated products and 30% of eriodictyol under the same catalytic conditions (Fig. 3). Thus, GmOMTs in clade A might prefer flavonoids with 2–3 carbon-carbon double bonds (C=C), for example, flavone and flavonol with 2–3 carbon-carbon single bonds (C–C; e.g., flavanone).

**Fig. 4:**
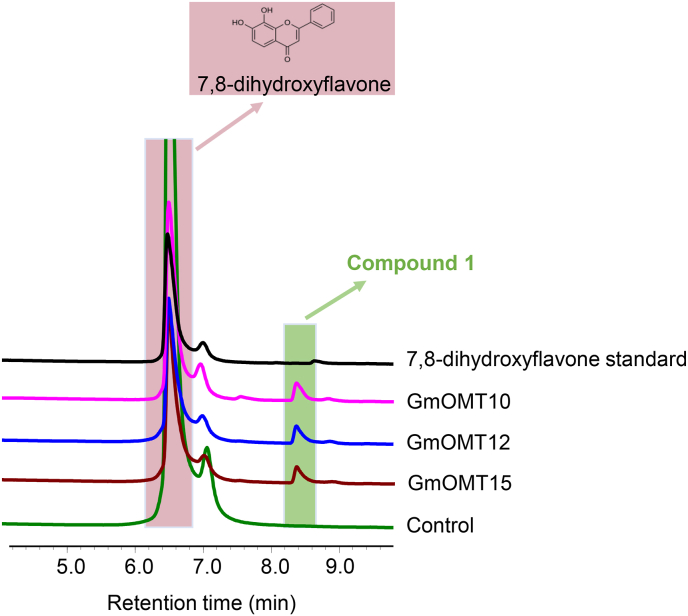
**Enzyme activities assay of GmOMT10, 12, and 15 toward 7,8-dihydroxyflavone as substrate**. HPLC analysis of the reaction products produced by incubating crude enzymes of GmOMT10, 12 and 15 with 7,8-dihydroxyflavone. Crude enzyme made from *E. coli* strain with empty pGEX-4T-1vector was used as a negative control. The newly generated peak is marked as compound 1.

The regiospecificity of OMTs from clade B was not as consistent as that of clade A. For example, MpOMT2 and ObF8OMT-1 are flavonoid 8-OH OMTs, and CrOMT2 and CrOM6 are 3′-OH/5′-OH and 4′-OH OMT, respectively. Five GmOMTs (4, 7, 10, 12, and 15) classified in this clade were tested using different flavonoids as substrates. Among them, GmOMT10, 12, and 15, exhibited catalytic activity toward 7, 8-dihydroxyflavone, resulting in a new peak (compound 1) in the HPLC and LC/MS analysis (Fig. 4 & Fig. S1). Because 7, 8-dihydroxyflavone has only two hydroxyls, 7-OH and 8-OH, only 8-OH methylation activities have been reported in this clade. We hypothesized that this novel compound is 7-hydroxy-8-*O*-methylflavonoid, an 8-OH methylation product of 7, 8-dihydroflavone. The other two GmOMTs, GmOMT4 and 7, exhibited no catalytic activity toward the tested flavonoids (Table 1), suggesting that they are non-flavonoid OMTs.

**Table 1:** Enzymatic activities of GmOMTs toward different flavonoids and caffeic acid as substrates. All the reactions were conducted under the same conduction. Numbers outside in the table represent the position of methylation. Conversion rate: , not detected; * <10%; 10%< ** <50%; *** >50%.

GmOMTs	Daidzein	Glycetein	Naringenin	Eriodictyol	Luteolin	Kaempferol	Quercetin	Caffeic acid	7,8-Dihydroxyflavone
GmOMT1	7(***)	7(***)	–	–	–	–	–	–	–
GmOMT2	–	–	–	–	4′(***)	4′(*)	–	–	–
GmOMT3	4′(***)	4′(***)	–	–	–	–	–	–	–
GmOMT4			–	–	–	–	–	–	–
GmOMT5	7(**) 4′(**)	7(**) 4′(*)	–	–	–	–	–	–	–
GmOMT6	–	–	–	3′(**)	3′(***)		3′(***)	3′(***)	–
GmOMT7	–	–	–	–	–	–	–	–	–
GmOMT8	–	–	4′(***)	4′(**)	–	–	–	–	–
GmOMT9	–	–	–	–	4′(**)	4′(**)	–	–	–
GmOMT10	–	–	–	–	–	–	–	–	8(**)
GmOMT11	–	–	–	3′(**)	3′(***)	–	3′(***)	3′(***)	–
GmOMT12	–	–	–	–	–	–	–	–	8(**)
GmOMT14	–	–	–	3′(**)	3′(***)	–	3′(***)	3′(***)	–
GmOMT15	–	–	–	–	–	–	–	–	8(**)
GmOMT16	7(***)	–	–	–	–	–	–	–	–
GmOMT17	–	–	7(**)	7(**)	7(***)	7(***)	7(***)	–	–
GmOMT18	–	–	–	3′(**)	3′(***)	–	3′(***)	3′(***)	–
GmOMT19	–	–	–	–	–	–	–	–	–
GmOMT20	–	–	–	–	–	–	–	–	–

**Fig. 5:**
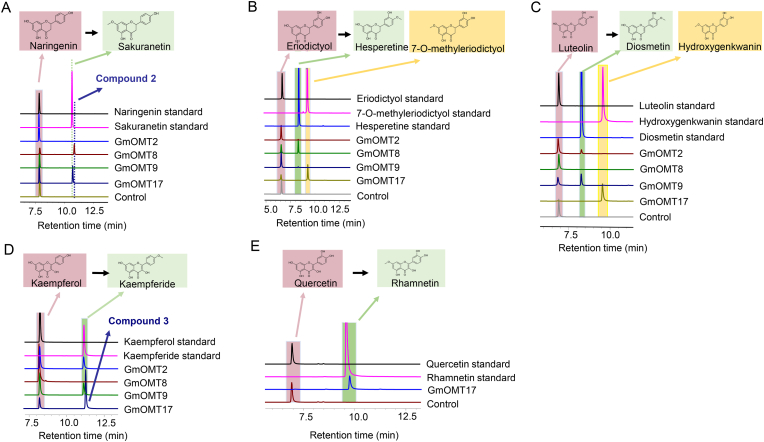
**Enzyme activities assay of GmOMT2, 8, 9, and 17 toward different flavonoid substrates**. HPLC analysis of the reaction products produced by incubating crude enzymes of GmOMT2, 8, 9, and 17 with naringenin (A), eriodictyol (B), luteolin (C), kaempferol (D), and quercetin (E) as substrate, respectively. Crude enzyme made from *E. coli* strain with empty pGEX-4T-1vector was used as a negative control. Authentic standards naringenin, sakuranetin, eriodictyol, 7-*O*-methyleriodictyol, hesperetine, luteolin, diosmetin, hydroxygenkwanin, kaempferol, kaempferide, quercetin, and rhamnetin were used for the detection and verification of the corresponding flavonoids products. The structures of the two novel products (compound 2 and 3) produced by GmOMT8 and 17, respectively, were further determined by 1H NMR. Compound 2 is confirmed as isosakuranetin (Fig. S3). Compound 3 is confirmed as rhamnocitrin (Fig. S5).

GmOMT8 was the only GmOMT in clade C1, the enzymatic assay indicated, that it could catalyze the 4′-OH methylation of naringenin and eriodictyol to compound 2 (isosakuranetin) (Fig. 5A & Figs. S2–3) and hesperetine (Fig. 5B). Other OMTs from this clade have been proven to be 4′-OMTs (MtIOMT5-7, GeHI4′OMT and LjHI4′OMT) (Fig. 2).

GmOMT2 and 9, in clade C2, were able to catalyze the 4′-OH methylation of luteolin and kaempferol to produce diosmetin (Fig. 5C) and kaempferide (Fig. 5D), respectively. Although GmOMT8 from clade C1, and GmOMT2 and 9 from clade C2, were all confirmed to be flavonoid 4′-OMT, they exhibited different substrate specificity. GmOMT8 was inclined to catalyze 2, 3-C-C flavonoids, converting naringin and eriodictyol into sakuranetin and hesperetine, but showed no detectable activity with 2, 3-C=C flavonoids (Fig. 5, Table 1); GmOMT2 and GmOMT9 favored methylating luteolin and kaempferol with 2, 3-C=C to produce diosmetin and kaempferide, and the catalytic activity of 2, 3-C-C flavonoids was extremely low (Fig. 5, Table 1). Thus, the presence of double bonds in flavonoids 2, 3 the position might significantly affect substrate recognition and catalysis.

GmOMT17 from clade C2 catalyzed the methylation of 7-OH of all selected flavonoids to produce the corresponding methylation products (naringenin to sakuranetin (Fig. 5A), eriodictyol to 7-*O*-methyleriodictyol (Fig. 5B), luteolin to hydroxygenkwanin (Fig. 5C), and kaempferol to compound 3 (rhamnocitrin) (Fig. 5D & S4-5) and quercetin to rhamnetin (Fig. 5E); however, the conversion rate varied for different substrates. GmOMT17 prefers flavones and flavonols rather than falvanones and flavanonols because the conversion rate of the former was significantly higher than that of the latter (Fig. 5, Table 1). The other two GmOMTs, GmOMT19 and 20, from clade C2 exhibited no catalytic activity toward the tested flavonoids (Table 1).

**Fig. 6:**
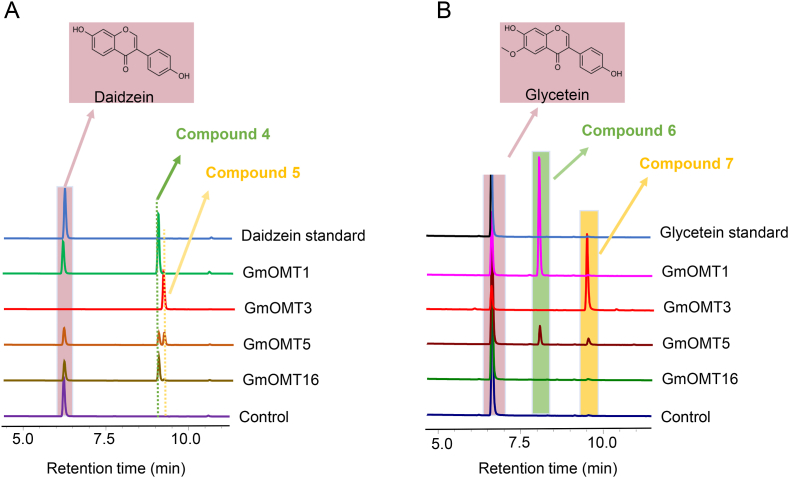
**Enzyme activities assay of GmOMT1, 3, 5, and 16 toward different flavonoid substrates**. HPLC analysis of the reaction products produced by incubating crude enzymes of GmOMT1, 3, 5, and 16 with daidzein (A) and glycetein (B) as the substrate, respectively. Crude enzyme made from *E. coli* strain with empty pGEX-4T-1vector was used as a negative control. Authentic daidzein and glycetein standards were used for the detection of the flavonoid products. The structures of the four novel products (compounds 4, 5, 6, and 7) produced by GmOMTs were further determined by 1H NMR. Compounds 4 and 5 are characterized as isoformononetin and formononetin, respectively (Figs. S7 and S12). Compounds 6 and 7 are characterized as 7-*O*-methylglycetein and 4′-*O*-methylglycetein, respectively (Figs. S9 and S14).

Except for SOMT2, OMTs from clade C3 were isoflavones methyltransferases, namely the isoflavones 7-OMTs MtIOMT1-3 and GeD7OMT and the isoflavones 4′-OMT MsI7OMT. Among the four GmOMTs clustered in this clade, GmOMT1 could catalyze daidzein and glycetein into compound 4 (isoformononetin) (Fig. 6A & Figs. S6–7) and compound 6 (7-*O*-methylglycitein) (Fig. 6B & Figs. S8–9), and GmOMT16 could only methylate daidzein (Fig. 6A & Fig. S10). GmOMT3 was identified as a 4′-OMT of isoflavones, which methylate daidzein and glycetein to compound 5 (formononetin) (Fig. 6A & Figs. S11–12) and compound 7 (4′-*O*-methylglycetein) (Fig. 6B, Figs. S13–14) respectively. However, GmOMT5, with very low substrate specificity, could methylate daidzein and glycetein at both 7- and 4′-OH (Fig. 6 & Figs. S15–16), and the conversion rate was very low (Table 1).

### 3.4 Summarizing conserved motifs for plant flavonoids OMTs

Thirty-eight FOMTs from different plants have been characterized previously (Table S3). The substrate specificities of these FOMTs and 15 GmOMTs characterized in this study are listed in Table S3. Most plant FOMTs belonged to the Leguminosae family, including *Glycine* max (16), *Medicago truncatula* (7), *Glycyrrhiza echinata* (2), *Medicago sativa* (1), and *Lotus japonicus* (1). The next genera was Lamiaceae, containing 12 FOMTs (*Ocimum basilicum* (7) and *Mentha x piperita* (5)), and Poaceae, containing three FOMTs (*Triticum aestivum* (1), *Oryza sativa* (1), and *Hordeum vulgare* (1)). The remaining species were *Chrysosplenium americanum* (three), *Catharanthus roseus* (two), *Citrus depressa* (two), *Solanum habrochaites* (two), *Arabidopsis thaliana* (one), and *Vanilla planifolia* (one) (Table S3). They were from 336 to 390 amino acids in length; 22–99% identical in the protein sequence; and catalyzed the methylation of flavonoids at 3-, 5-, 6-, 7-, 8-, 3’-, 4′-OH and isoflavonoids at 7-, 4′-OH.

**Fig. 7:**
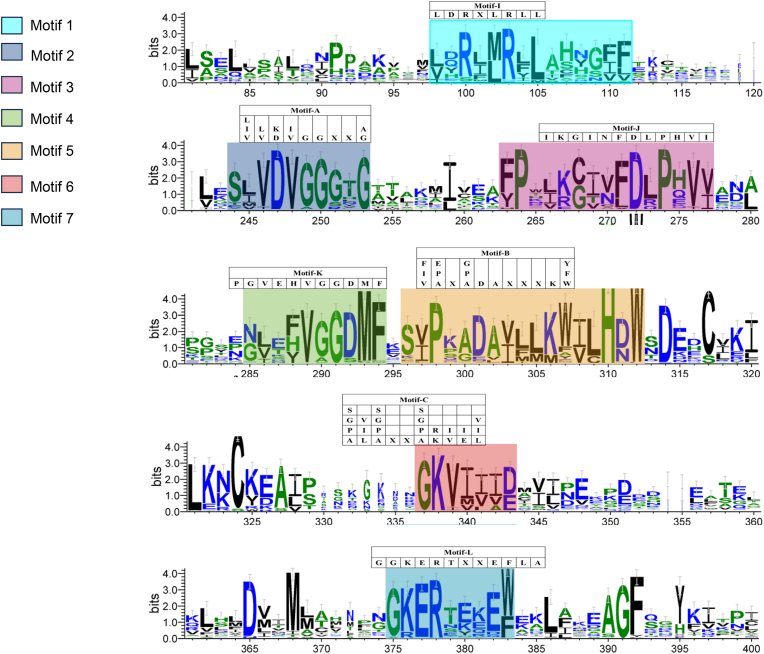
**Conserved motifs analysis of plant flavonoid OMTs**. Fifty-three plant flavonoid OMTs, comprising the 15 GmOMTs characterized in this study and 38 previously reported OMTs, were subjected to sequence alignment, and the converted motifs were generated using WebLogo. The seven conserved motifs 1–7 proposed in this study are marked in different colors. The corresponding motifs A, B, C, I, J, K, and L, proposed by Joshi and Chiang [31], are listed above the logo at the corresponding site

Joshi and Chiang proposed conserved motifs (Motif A, B, C, I, J, K, and L) for plant OMTs [31]. In this study, more than 50 OMTs used flavonoids as substrates. Thus, we posited that summarizing the conserved motifs specific to FOMTs would be possible. Thus, this study conducted multiple sequence alignment of all 53 characterized FOMTs, and seven novel conserved motifs were proposed (motifs 1–7) (Fig. 7).

The first highly conserved Motif 1 with (L/V) (D/Q/Y/H) (R/S) X(M/L) RXLXXX(G/S/Z) (F/I/V) (F/L/V) had six more residues than the motif I proposed by Joshi and Chiang (Fig. 7). Motif 1 described the conserved sequences of the FOMTs more precisely. Motif 2, with (S/T/E) (I/L/M/V) (V/I) D V G G (G/S/R) X G as a consensus sequence, corresponds to Motif A proposed by Joshi and Chiang (Fig. 7), with small modifications. Motif 2 had one more residue than motif A. The residue D/K at the fourth position in Motif A changed to residue D in Motif 2 in the absence of lysine (K) at this position in FOMTs, which was the same as the residue G/A at the end position in Motif A being replaced by residue G in Motif 2. Motif 3 had the consensus sequence (F/Y) P X (L/I/V) (K/R/E) (C/G) (I/T/V) X (F/L) D (L/Q/R) P X (V/I) (V/I), which was present in 52 of the 53 characterized FOMTs with 0–1 mismatches (Fig. 7). Motif 3 was three residues longer than Motif J and was more accurate for FOMTs. The situation of FOMTs could not be well summarized by residues I, G, N, H, and I in the first, third, fifth, tenth, and twelfth positions of Motif J. Motif 4 was similar to Motif K, but the case of Motif 4 could not be well summarized by residues G, V, E, and H in the first to fourth positions in Motif K. This motif was present in 98% of FOMTs with 0–1 mismatches. Residues Asp and Gly at the fourth and tenth positions in motif 2, Pro at the second and twelfth positions in motif 3, and Gly at the seventh position in motif 4 were 100% conserved in all 53 FOMT sequences, suggesting that they play a critical role in maintaining the activity of FOMTs. The consensus sequence of Motif 5 was S(V/I) PX(A/G) DA(V/I) (L/F/M) (L/M) (W/F) (I/V) (L/C) H (D/N) W, which contained five more residues than motif B (Fig. 7). Residues A/P/E in the second position of Motif B were replaced with residue P in Motif 5. Residues A/P/G in the fourth position of Motif B did not contain residue P in Motif 5. Motif 6, with seven residues, had a consensus G K V (I/M/V) (I/V/L) (I/V/A) (D/E) that was also present in 51 of 53 FOMTs with zero mismatches (Fig. 7). Motif 6 occupied a part of Motif C, as suggested by Joshi, and underwent an accurate modification specialized for FOMTs. Although highly conserved in the SAM-dependent MTs selected by Joshi, the first to fifth residues in Motif C were still deleted in Motif 6 because they are not conserved in FOMTs. Residues A/P/G/S in the first position of motif 6 replaced residue G in motif C. In addition, the deletion of residue E and the presence of residues V/M resulted in motif 6 having residues I/V/M instead of residues E/I in the fourth position. The consensus sequence for Motif 7 was G (K/R) E RX (E/K/Y) XE(W/F), which was present in all 53 characterized FOMTs with 0–1 mismatches (Fig. 7). Motif 7 had three fewer residues than Motif L; the N-terminal residue of Motif 7 was W or F with W being dominant; and in Motif L, this position was only residue F. Collectively, we found that although the seven motifs were highly conserved among OMTs, the novel proposed motifs 1–7 of FOMTs had small modifications compared with the literature. The summary and proposal of conserved motifs specific to flavonoids might facilitate the discovery and functional prediction of OMTs in plants.

## 4 Conclusion

*Glycine* max (soybean) contains various flavonoids, most of which are in methylated form; for example, isorhamnetin is 3′-methylated quercetin, afromosin, and formononetin are 4′-methylated glycetein and daidzein. Therefore, *G.* max may be ideal for studying flavonoid OMTs. In this study, using gene screening at the genome-scale level, we obtained 22 potential OMTs from *G. max*, 19 of which were cloned successfully, and 15 were able to catalyze the methylation of flavonoids. Regarding methylation sites, these OMTs could catalyze isoflavone 7-/4′-OH and flavonoid 7-/8-/3'-/4′-OH, covering almost all the common hydroxyl sites of flavonoids. Notably, we are the first to observe that GmOMT2 and 9 are OMTs that can catalyze the methylation of luteolin 4′-OH to produce diosmetin. The systematic characterization of the GmOMTs expands the knowledge of the biosynthesis of methylated flavonoids in *G*. max and of the enzyme library of flavonoid OMTs, facilitating the biosynthesis of methylated flavonoids via synthetic biology. In addition, we summarized seven novel motifs specific to FOMTs; they are generally as conserved as the OMT motifs in the literature but have many modifications specified for FOMT. These novel motifs will facilitate the discovery of novel flavonoid OMTs.

## CRediT authorship contribution statement

**Bingtong Feng:** Conceptualization, Investigation, Writing – original draft. **Yuguo Jiang:** Conceptualization, Investigation. **Xiaodong Li:** Investigation. **Yan Wang:** Investigation. **Ziyu Ren:** Investigation. **Jian Lu:** Supervision. **Xing Yan:** Project administration, Supervision. **Zhihua Zhou:** Project administration, Conceptualization, Writing – review & editing, Supervision. **Pingping Wang:** Conceptualization, Writing – original draft, Writing – review & editing, Supervision.

## Declaration of competing interests

The authors have no interests to declare.
