# Supplementary material for: Systemically functional characterization of regiospecific flavonoid O-methyltransferases from Glycine max
Source: Synth Syst Biotechnol. 2024 Mar 15;9(2):340–8. doi: 10.1016/j.synbio.2024.03.009 (PMC10972763; doi:10.1016/j.synbio.2024.03.009)
Supplement: Multimedia component 1 [file mmc1.docx]

**Supplementary Information for**

**Systemically functional characterization of regiospecific flavonoid O-methyltransferases from *Glycine max***

Feng et al.,

**Table S1. Primers used in this sdudy.**

| Primer | Sequence (5’ to 3’) |
| --- | --- |
| GmOMT1-F | AAGATCAAACTTCGTTTGCAATT |
| GmOMT1-R | TAAATTAATCAAAACATTAATGT |
| GmOMT2-F | TAAGCAAACATAACTTGCAGTA |
| GmOMT2-R | GACATACACATCATAAAAATGT |
| GmOMT3-F | CAAAAAAAGAATCAGATGCAATT |
| GmOMT3-R | AATAAAAAATAAAACATAATTAT |
| GmOMT4-F | ACCCCAAACCTAGCTACG |
| GmOMT4-R | ATCCTCGACAAGCAGCTC |
| GmOMT5-F | GATCAAATTTCGTTTGCAATT |
| GmOMT5-R | TAAATAAATTGCAGCATTAGTAT |
| GmOMT6-F | CTTCACAGAGCAAAGACAC |
| GmOMT6-R | CTCCTATAAGGGTTGGCAC |
| GmOMT7-F | TAGTTCAATTAAATTTGCAAAAC |
| GmOMT7-R | TGCCACCCATCATTTCAC |
| GmOMT8-F | CCTTCGAAAACCACTCACTC |
| GmOMT8-R | CAAGATGAAACCGTAGTACACA |
| GmOMT9-F | CCAATGCTGGTCATGGAAAC |
| GmOMT9-R | ACCAGAAGTTGAGACAATGC |
| GmOMT10-F | GCTTAAACATCTTCTCGCC |
| GmOMT10-R | GAATGATAGAGTGCTGGCA |
| GmOMT11-F | CAGTTCCAATCTAAAATCAACC |
| GmOMT11-R | GAATCCACGCCAAAGAAC |
| GmOMT12-F | ATACTTCCTTAAAGCAGAAAAATT |
| GmOMT12-R | GGTCCTTCTCCTATATACCAC |
| GmOMT13-F | AGCTAGAGTTAAGAGAGGAG |
| GmOMT13-R | GGGTAATTTTTGGTGGATATATT |
| GmOMT14-F | AAGCAAACCAAACATCTTGAATC |
| GmOMT14-R | ATGAATCCATGCCAAAGAAC |
| GmOMT15-F | AGTTTAGGCAGCAAATTATGG |
| GmOMT15-R | GCTGGCACATCTCATTAAC |
| GmOMT16-F | CAACAAAAATTTACAAGCAACT |
| GmOMT16-R | TTGGAAAGCGTTCGTTGACA |
| GmOMT17-F | TGTCTTCCATGGATAACC |
| GmOMT17-R | CTAAGGATAGAGCTCAATTAG |
| GmOMT18-F | CTTAGGCAAACCAAAGCTCC |
| GmOMT18-R | CCACAAGTAGAAAGAGTCTCAAG |
| GmOMT19-F | CGCTTGCAGTAGTAATGGA |
| GmOMT19-R | CCTAAGCATTAAGGATAGACC |
| GmOMT20-F | GCAAATATCACTTGCAGTACTA |
| GmOMT20-R | AACAATGCCAATATGCCATCT |
| GmOMT21-F | GGCTTCAAATAATGGCCG |
| GmOMT21-R | TGCCACGATTTTCAACAAAC |
| GmOMT22-F | TTGCAGTAATGGCTTCAATG |
| GmOMT22-R | TATTCAACTCACAACACGC |

**Table S2. Candidate OMTs annotated from the genome of *Glycine max*.**

| Gene name | Protein ID in Soycyc 8.0 | Sequence length | Identity with *SOMT2* |
| --- | --- | --- | --- |
| GmOMT1 | Glyma.18G267900.1 | 353 | 74.1 |
| GmOMT2 | Glyma.20G213700.1 | 354 | 53.6 |
| GmOMT3 | Glyma.08G248000.1 | 354 | 73.0 |
| GmOMT4 | Glyma.06G286700.1 | 355 | 39.9 |
| GmOMT5 | Glyma.18G267800.1 | 354 | 77.2 |
| GmOMT6 | Glyma.04G227700.1 | 365 | 26.5 |
| GmOMT7 | Glyma.06G286200.1 | 359 | 41.6 |
| GmOMT8 | Glyma.13G173300.1 | 365 | 48.1 |
| GmOMT9 | Glyma.10G176500.1 | 354 | 54.7 |
| GmOMT10 | Glyma.14G201100.1 | 358 | 43.0 |
| GmOMT11 | Glyma.06G137300.1 | 365 | 26.3 |
| GmOMT12 | Glyma.06G286600.1 | 352 | 42.8 |
| GmOMT13 | Glyma.11G256500.1 | 366 | 30.1 |
| GmOMT14 | Glyma.06G137100.1 | 365 | 26.8 |
| GmOMT15 | Glyma.14G200900.1 | 358 | 42.7 |
| GmOMT16 | Glyma.18G269600.1 | 382 | 64.0 |
| GmOMT17 | Glyma.20G213600.1 | 354 | 52.5 |
| GmOMT18 | Glyma.06G137200.1 | 366 | 26.5 |
| GmOMT19 | Glyma.09G094400.1 | 353 | 51.7 |
| GmOMT20 | Glyma.10G176600.1 | 355 | 53.9 |
| GmOMT21 | Glyma.18G267500.1 | 359 | 73.9 |
| GmOMT22 | Glyma.10G176700.1 | 354 | 54.7 |

**Table S3. Characterized plant flavonoid OMTs.** Fifteen GmOMTs characterized in this study and 38 previous reported plant FOMTs were summarized.

| **Name** | **Origin** | **Gene ID** | **Year** | **length** | **site** |
| --- | --- | --- | --- | --- | --- |
| **CaOMT1** | *Chrysosplenium americanum* | P59049 | 1998 | 343 | 3' |
| **CaOMT2** | *Chrysosplenium americanum* | Q42653 | 1998 | 343 | 3' |
| **pFOMT3’** | *Chrysosplenium americanum* | Q42654 | 1996 | 343 | 3' |
| **CiOMT2** | *Citrus reticulata* | ADK97702 | 2020 | 366 | 73' |
| **GmOMT6** | *Glycine max* | - | This study | 365 | 3' |
| **GmOMT11** | *Glycine max* | - | This study | 365 | 3' |
| **GmOMT14** | *Glycine max* | - | This study | 365 | 3' |
| **GmOMT18** | *Glycine max* | - | This study | 366 | 3' |
| **AtOMT1** | *Arabidopsis thaliana* | U70424 | 1998 | 363 | 3' |
| **TaOMT2** | *Triticum aestivum* | Q38J50 | 2006 | 356 | 3' |
| **VanOMT3** | *Vanilla planifolia* | ABD61228 | 2006 | 359 | 3' |
| **CdFOMT5** | *Citrus depressa* | BAU51794 | 2016 | 353 | 3567 |
| **MpOMT3** | *Mentha x piperita* | AAR09601 | 2004 | 364 | 3’ |
| **ShMOMT1** | *Solanum habrochaites* | ADZ76433 | 2011 | 355 | 3' |
| **ShMOMT3** | *Solanum habrochaites* | AGK26768 | 2012 | 361 | 3 |
| **CrOMT2** | *Catharanthus roseus* | Q8GSN1 | 2003 | 348 | 3' |
| **MpOMT4** | *Mentha x piperita* | AAR09602 | 2004 | 343 | 4‘ |
| **ObFOMT3** | *Ocimum basilicum* | AFU50297 | 2012 | 336 | 64' |
| **ObFOMT5** | *Ocimum basilicum* | AFU50299 | 2012 | 336 | 64' |
| **CrOMT6** | *Catharanthus roseus* | AAR02419 | 2004 | 359 | 4' |
| **GmOMT8** | *Glycine max* | - | This study | 365 | 4' |
| **MtIOMT7** | *Medicago truncatula* | ABD83946 | 2006 | 363 | 74’ |
| **GmOMT2** | *Glycine max* | - | This study | 354 | 4' |
| **GmOMT9** | *Glycine max* | - | This study | 354 | 4' |
| **SOMT2** | *Glycine max* | C6TAY1 | 2005 | 358 | 4' |
| **GeD7OMT** | *Glycyrrhiza echinata* | Q84KK5 | 2003 | 357 | 7 |
| **MsI7OMT** | *Medicago sativa L* | AAC49928 | 1998 | 352 | 7 |
| **MtIOMT1** | *Medicago truncatula* | AAY18582 | 2006 | 352 | 7 |
| **MtIOMT2** | *Medicago truncatula* | ABD83942 | 2006 | 357 | 74‘ |
| **MtIOMT3** | *Medicago truncatula* | ABD83943 | 2006 | 357 | 74’ |
| **MtIOMT4** | *Medicago truncatula* | DQ419912 | 2006 | 361 | 7 |
| **GmOMT1** | *Glycine max* | - | This study | 353 | 7 |
| **GmOMT5** | *Glycine max* | - | This study | 354 | 74' |
| **GmOMT16** | *Glycine max* | - | This study | 355 | 7 |
| **OsNOMT1** | *Oryza sativa* | BAM13734 | 2012 | 378 | 7 |
| **MpOMT1A** | *Mentha x piperita* | AAR09598 | 2004 | 344 | 7 |
| **MpOMT1B** | *Mentha x piperita* | AAR09599 | 2004 | 344 | 7 |
| **ObFOMT1** | *Ocimum basilicum* | AFU50295 | 2012 | 340 | 7 |
| **ObFOMT2** | *Ocimum basilicum* | AFU50296 | 2012 | 340 | 7 |
| **F1-OMT1** | *Hordeum vulgare* | CAA54616 | 1998 | 390 | 7 |
| **GmOMT17** | *Glycine max* | - | This study | 354 | 7 |
| **GmOMT10** | *Glycine max* | - | This study | 358 | 8 |
| **GmOMT15** | *Glycine max* | - | This study | 358 | 8 |
| **GmOMT12** | *Glycine max* | - | This study | 352 | 8 |
| **MpOMT2** | *Mentha x piperita* | AAR09600 | 2004 | 366 | 8 |
| **ObF8OMT-1** | *Ocimum basilicum* | AGQ21572 | 2013 | 359 | 8 |
| **GeHI4'OMT** | *Glycyrrhiza echinata* | Q84KK6 | 2003 | 367 | 4' |
| **MtIOMT5** | *Medicago truncatula* | AAY18581 | 2006 | 364 | 4’ |
| **LjHI4'OMT** | *Lotus japonicus* | Q84KK4 | 2003 | 365 | 4’ |
| **MtIOMT6** | *Medicago truncatula* | ABD83945 | 2006 | 357 | 74‘ |
| **GmOMT3** | *Glycine max* | - | This study | 354 | 4' |
| **ObFOMT4** | *Ocimum basilicum* | AFU50298 | 2012 | 336 | 6 |
| **ObFOMT6** | *Ocimum basilicum* | AFU50300 | 2012 | 336 | 64' |

**
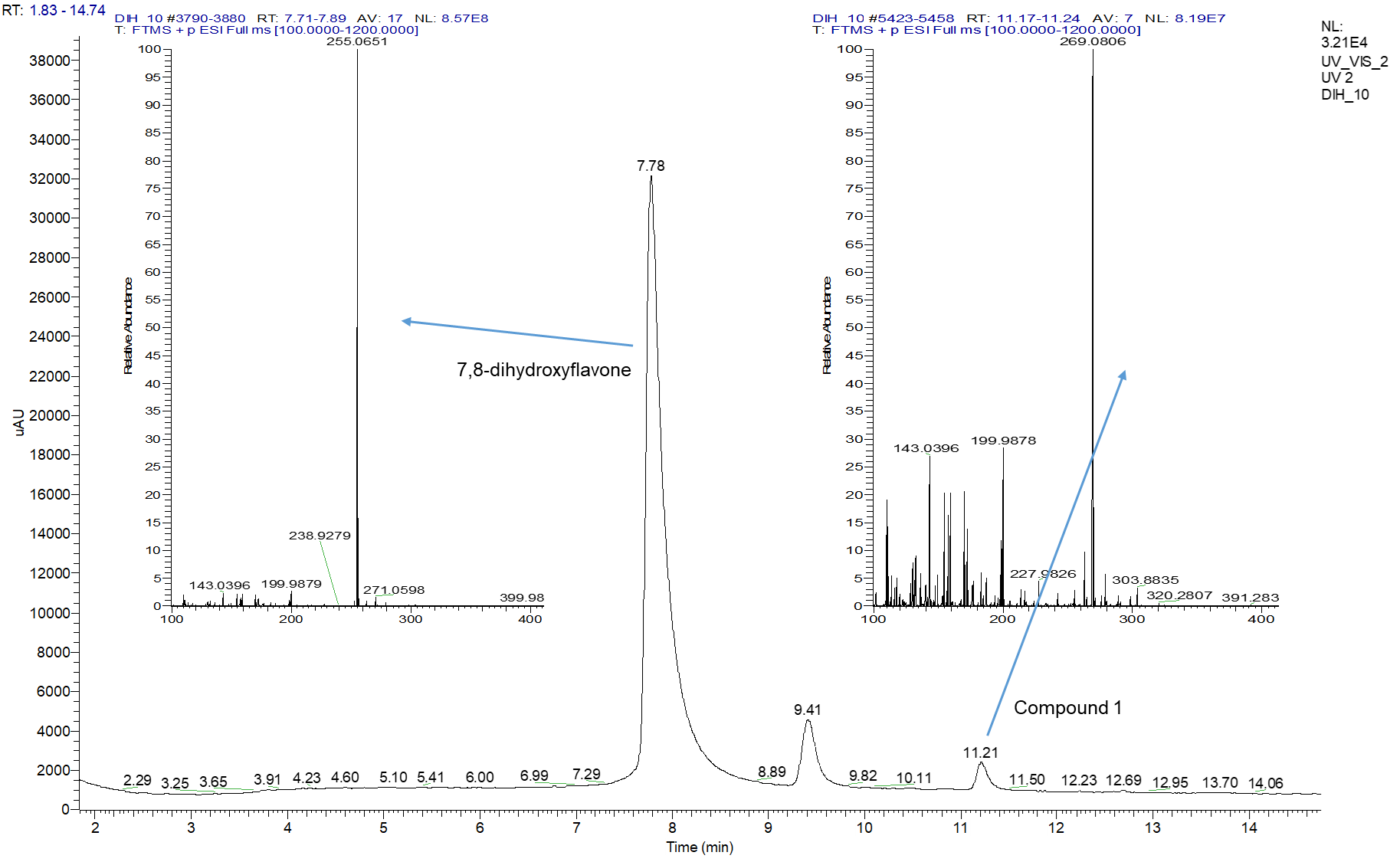
Figure S1** Mass spectrometry analysis of the reaction product compound 1 catalyze by GmOMT10.


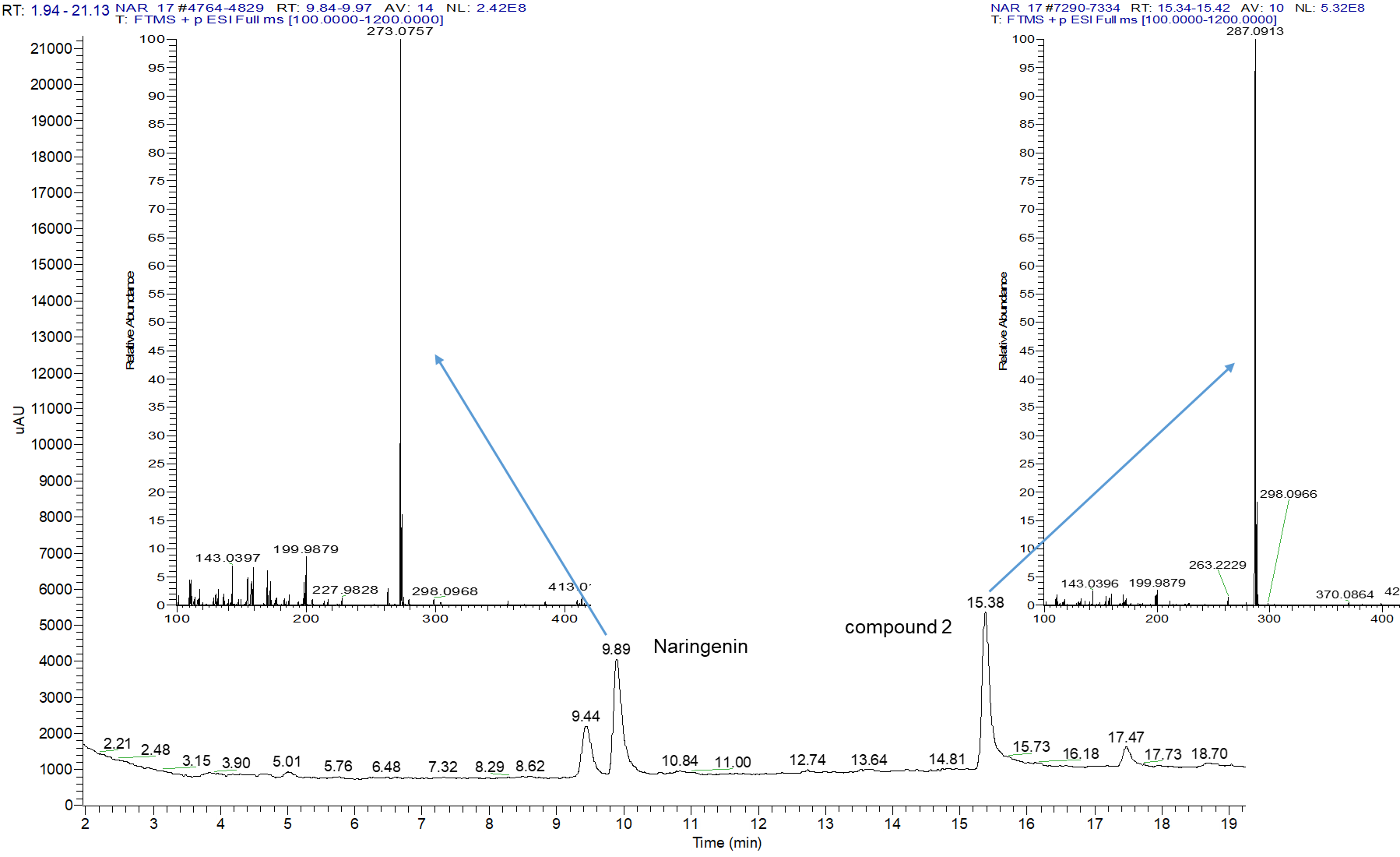


**Figure S2** Mass spectrometry analysis of the reaction product compound 2 catalyze by GmOMT8.


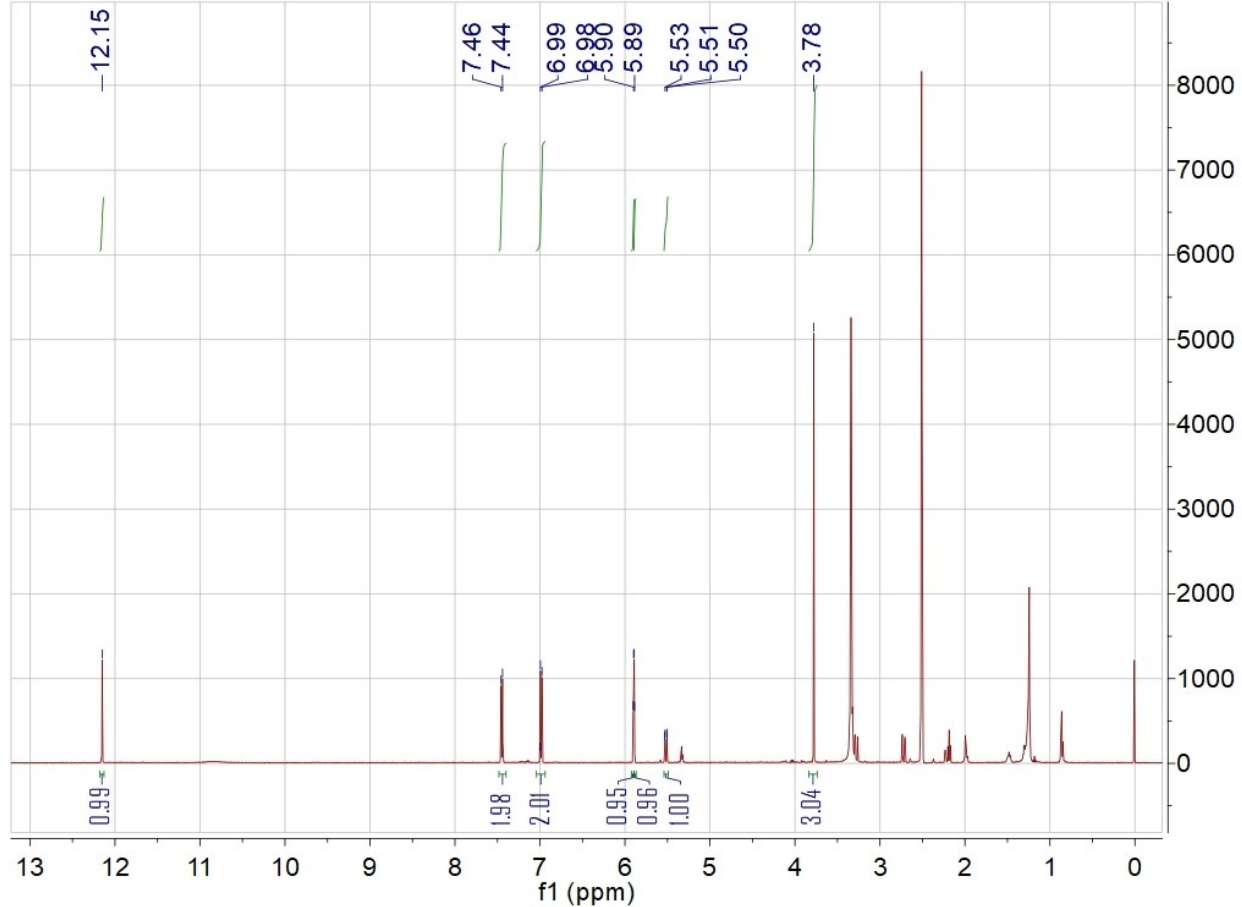


**Figure S3** 1HNMR analysis of compound 2.

1H NMR (400 MHz, in DMSO-d6) δ 3.78 (3H, s, 4’-OCH3), 5.52 (1H, dd, 3-H), 5.89 (1H, d, 8-H), 5.9 (1H, d, 6-H), 6.99 (2H, d, 3',5'-H), 7.45 (2H, d, 2',6'-H), 12.15 (1H, s, 2-H).


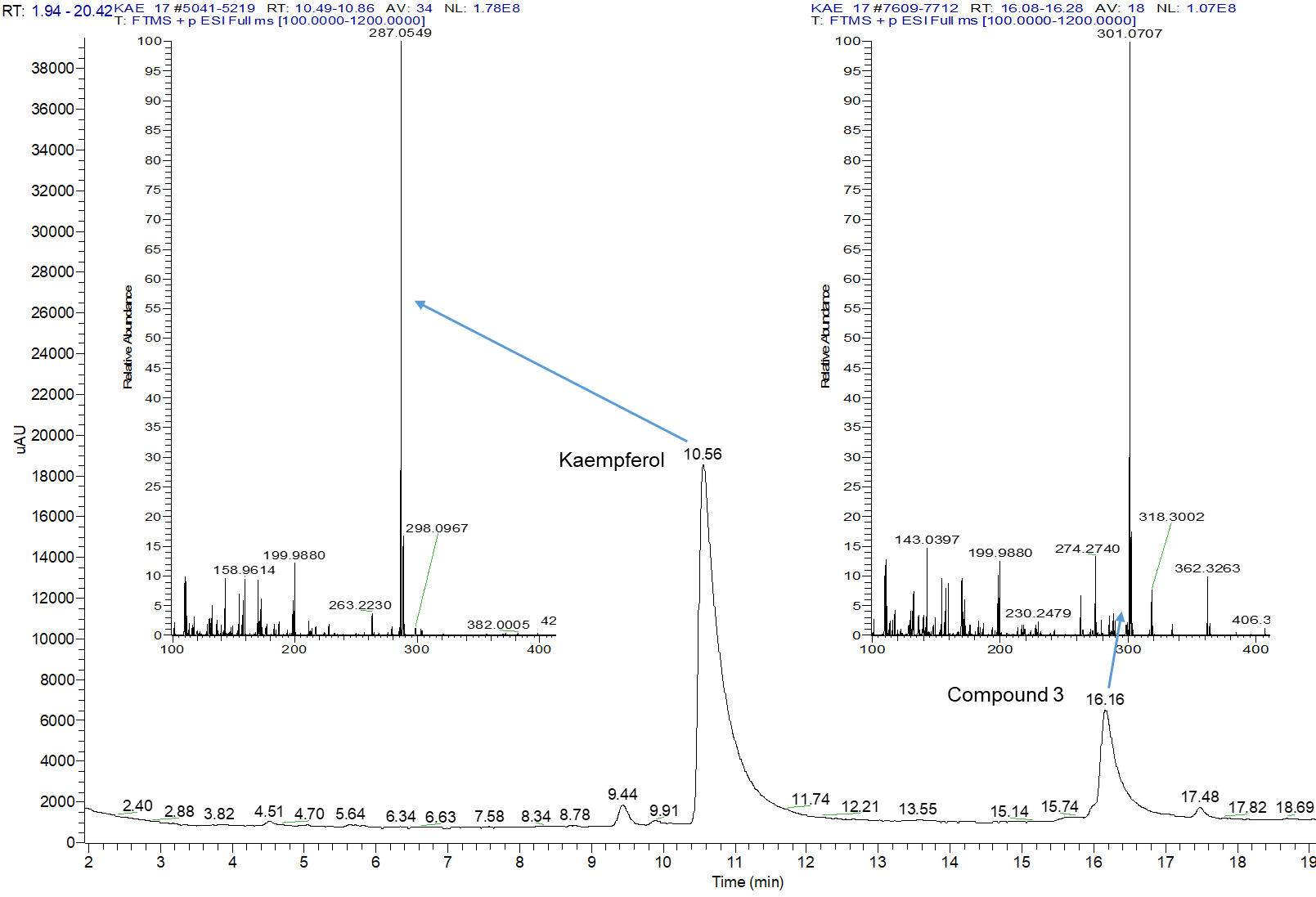


**Figure S4** Mass spectrometry analysis of the reaction product compound 3 catalyze by GmOMT17.


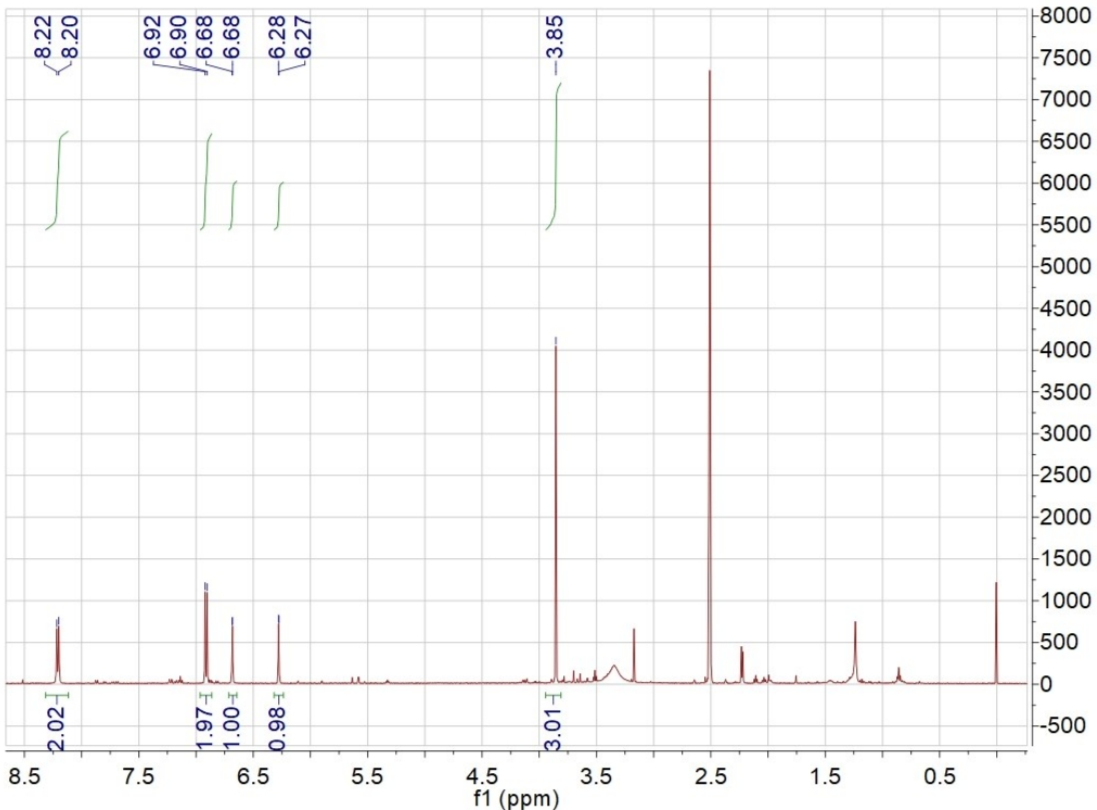


**Figure S5** 1HNMR analysis of compound 3.

1H NMR (400 MHz, in DMSO-d6) δ 3.85 (3H, s, 7-OCH3), 6.27 (1H, d, 8-H), 6.68 (1H, d, 6-H), 6.91 (2H, d, 3',5'-H), 8.21 (2H, d, 2',6'-H).

**
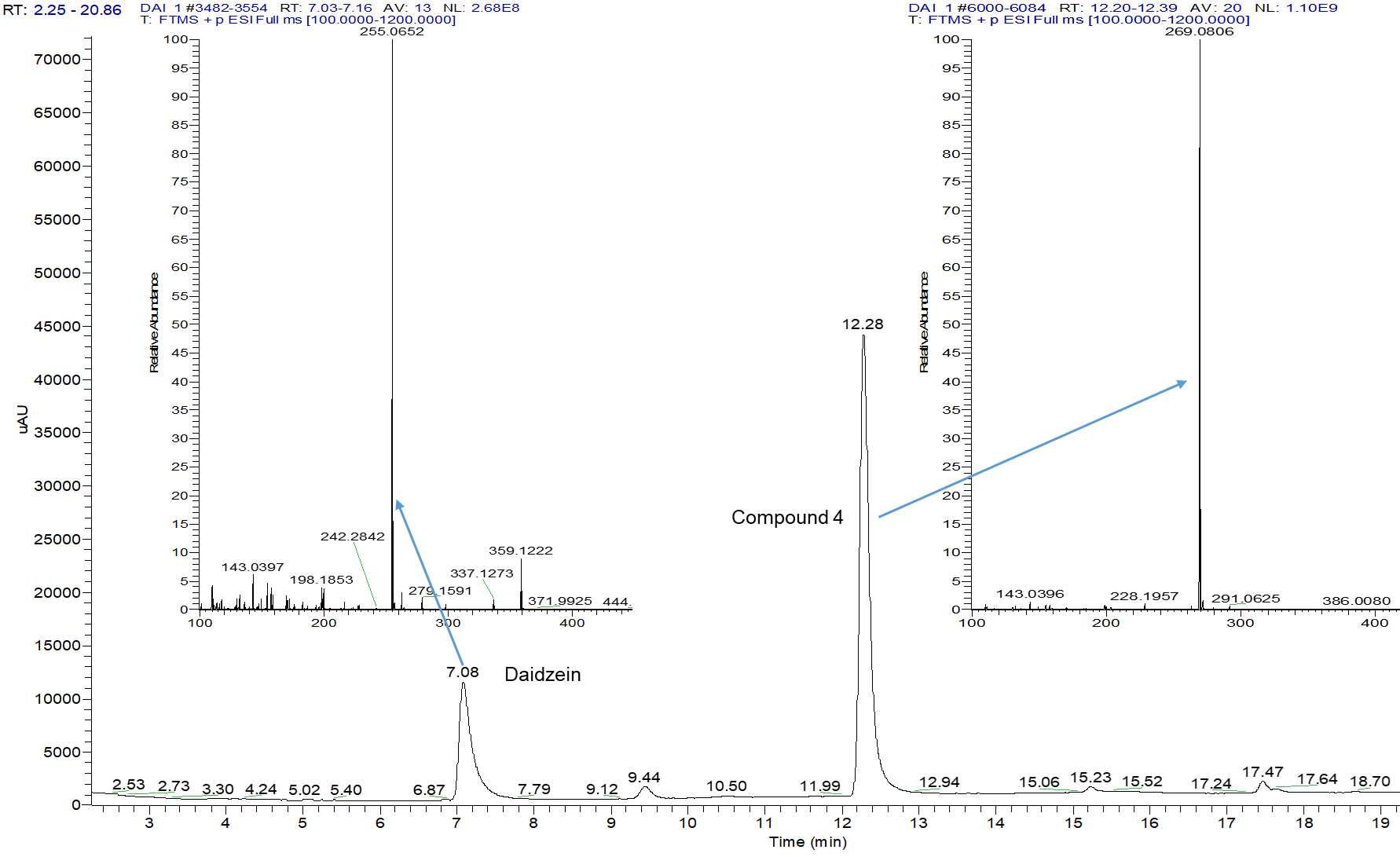
**

**Figure S6** Mass spectrometry analysis of the reaction product compound 4 catalyze by GmOMT1.


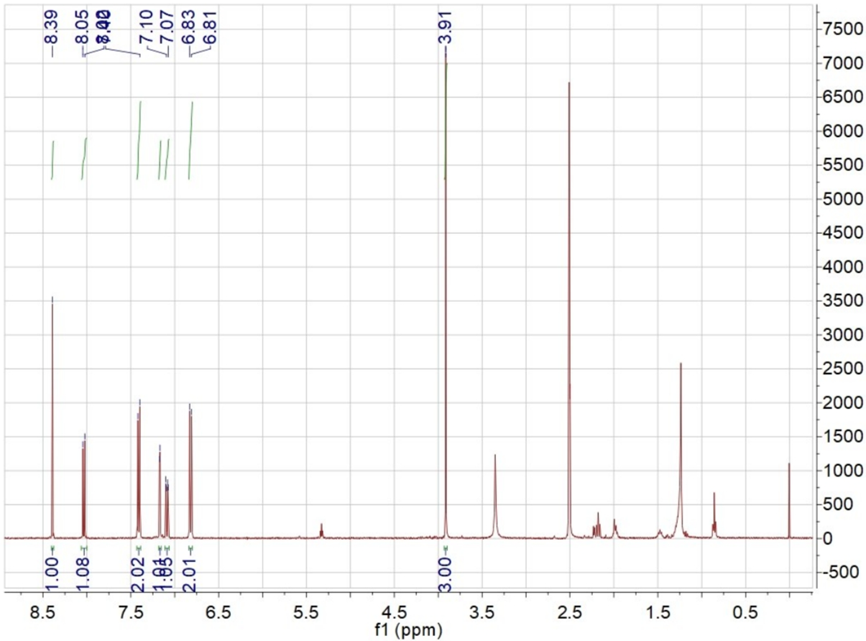


**Figure S7** 1HNMR analysis of compound 4.

1H NMR (400 MHz, in DMSO-d6) δ 3.91 (3H, s, 7-OCH3), 6.82 (2H, d, 3’,5’-H), 7.09 (1H, dd, 6-H), 7.17 (1H, d, 8-H), 7.41 (2H, d, 2',6'-H), 8.04 (1H, d, 5-H), 8.39 (1H, s, 2-H).


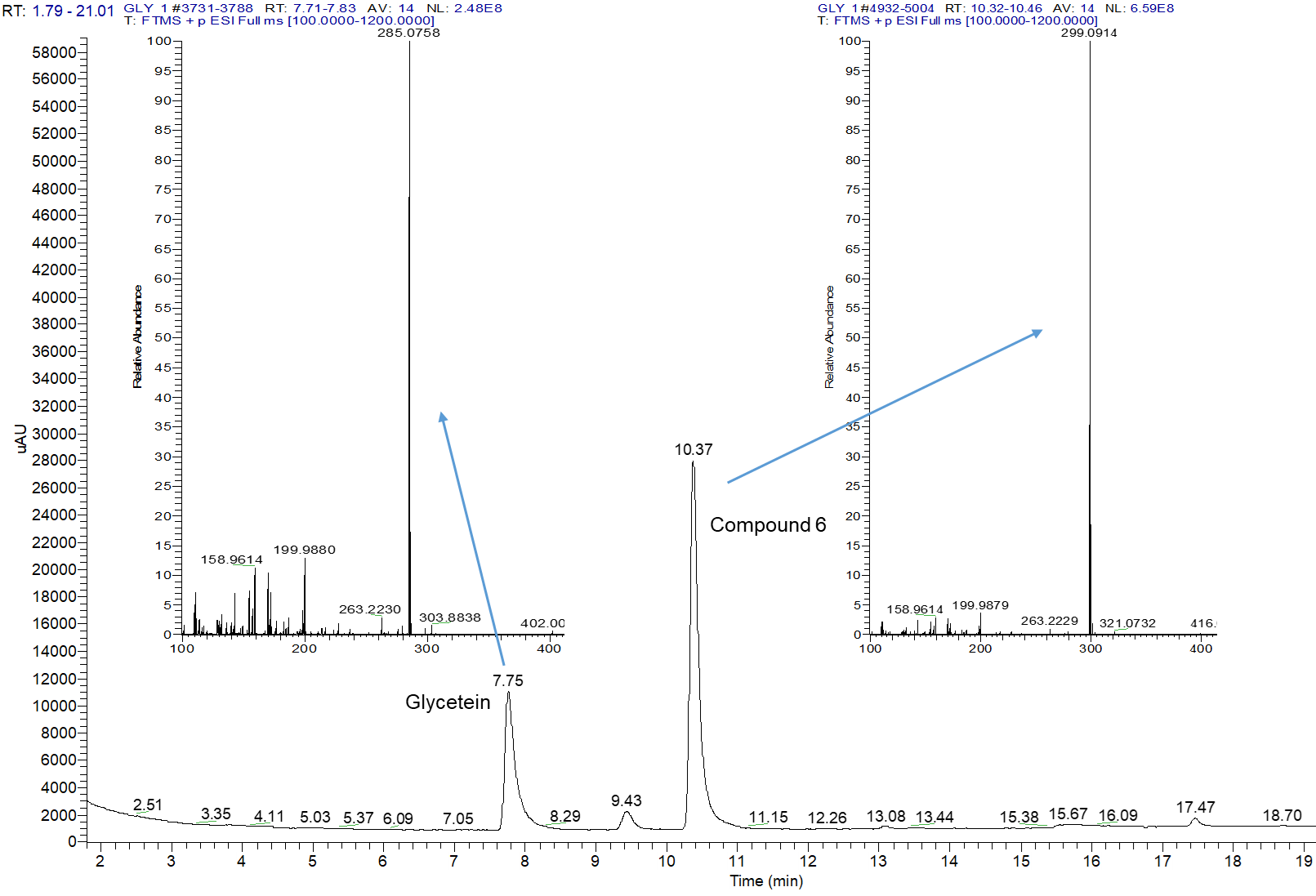


**Figure S8** Mass spectrometry analysis of the reaction product compound 6 catalyze by GmOMT1.


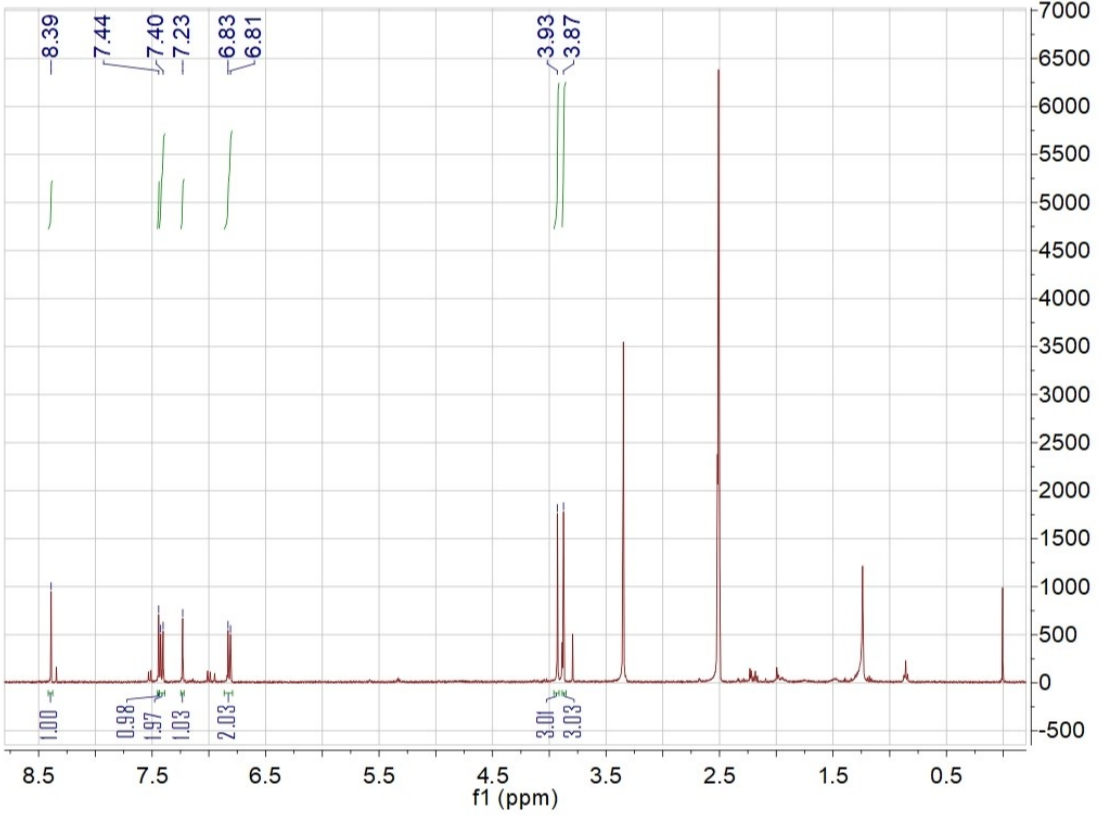


**Figure S9** 1HNMR analysis of compound 6.

1H NMR (400 MHz, in DMSO-d6) δ 3.87 (3H, s, 6-OCH3), 3.93 (3H, s, 7-OCH3), 6.82 (2H, d, 3',5'-H), 7.23 (1H, s, 8-H), 7.41 (2H, d, 2',6'-H), 7.44 (1H, d, 5-H), 8.39 (1H, s, 2-H).


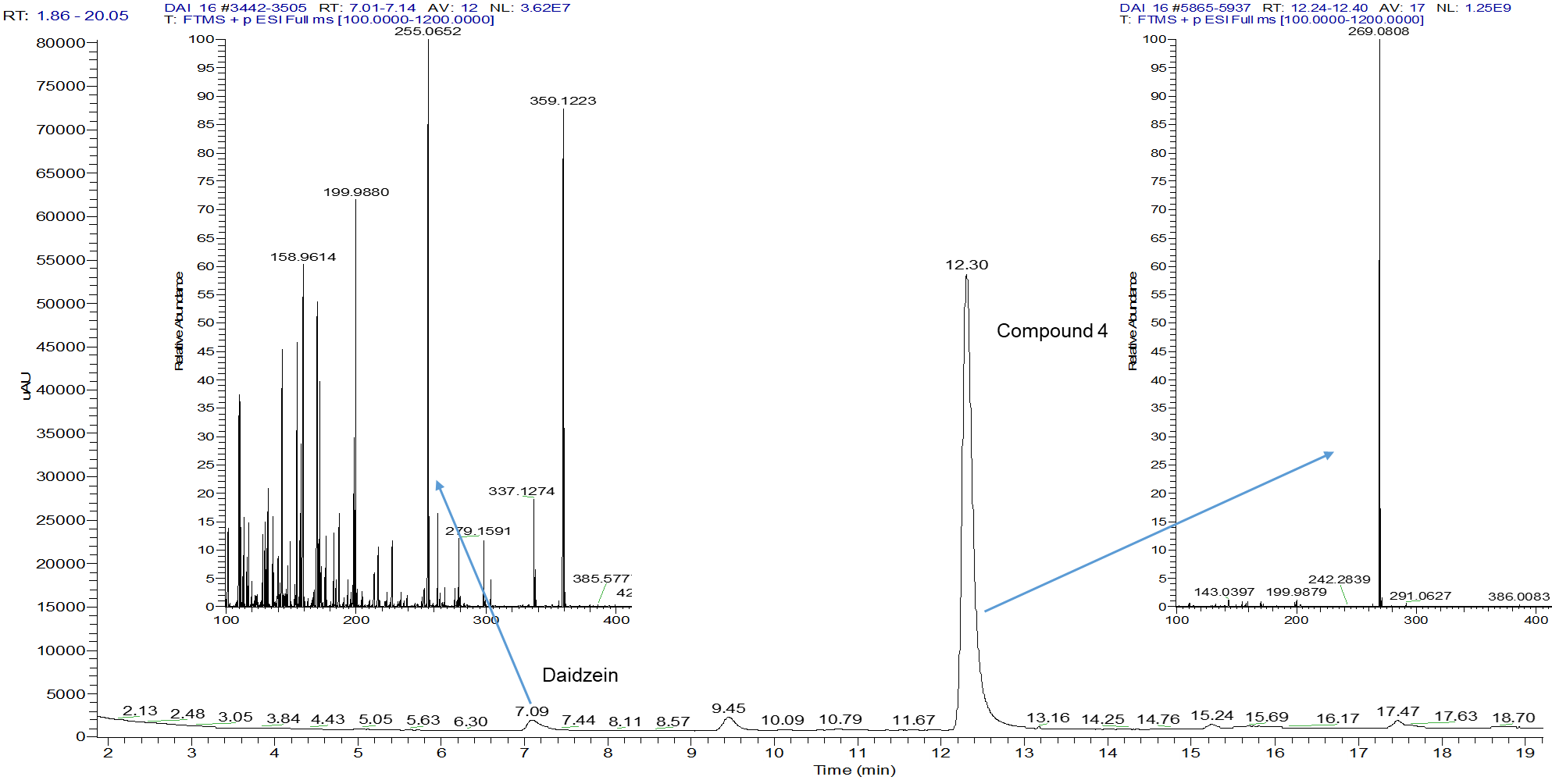


**Figure S10** Mass spectrometry analysis of the reaction product compound 4 catalyze by GmOMT16.


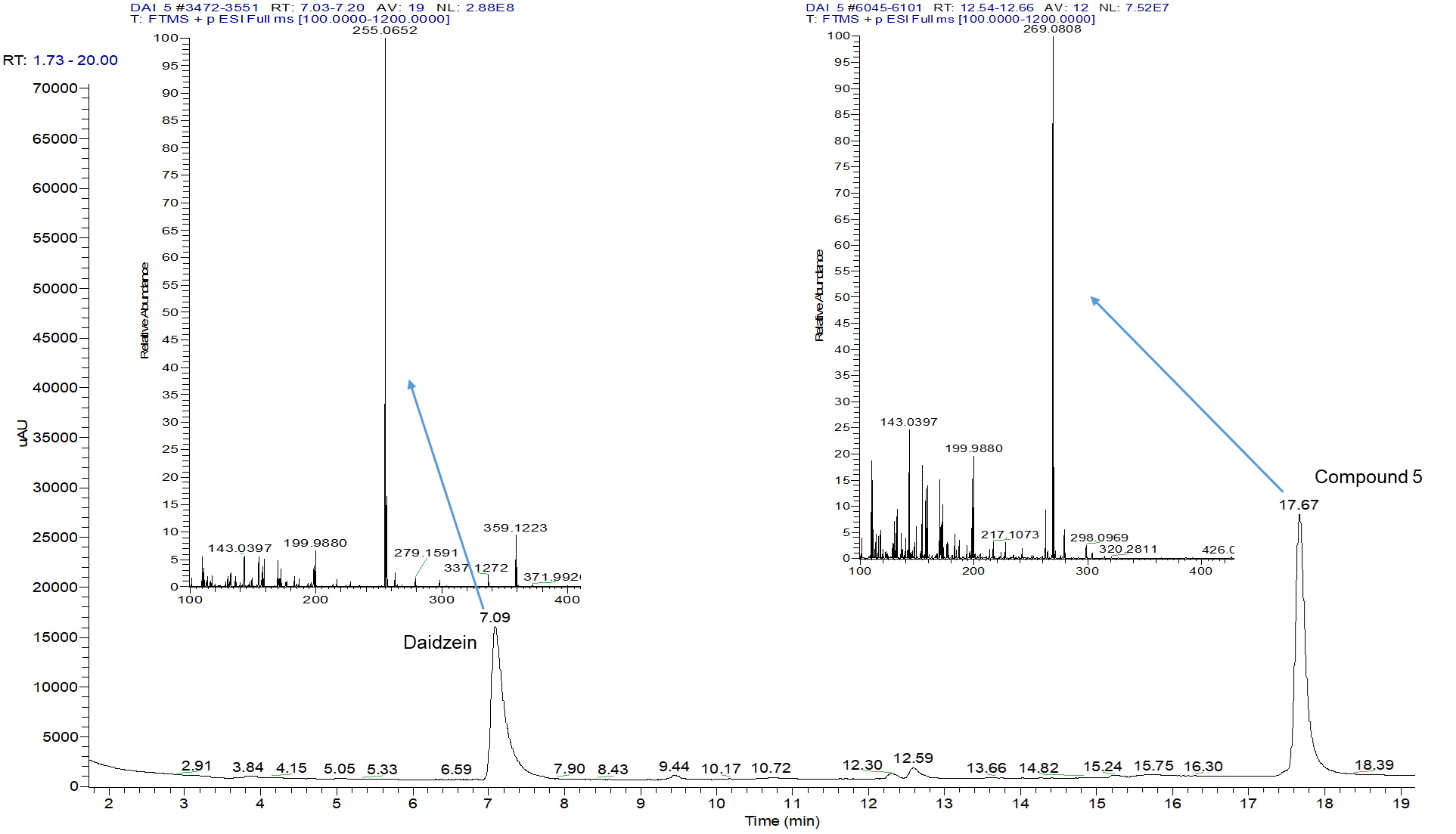


**Figure S11** Mass spectrometry analysis of the reaction product compound 5 catalyze by GmOMT3.


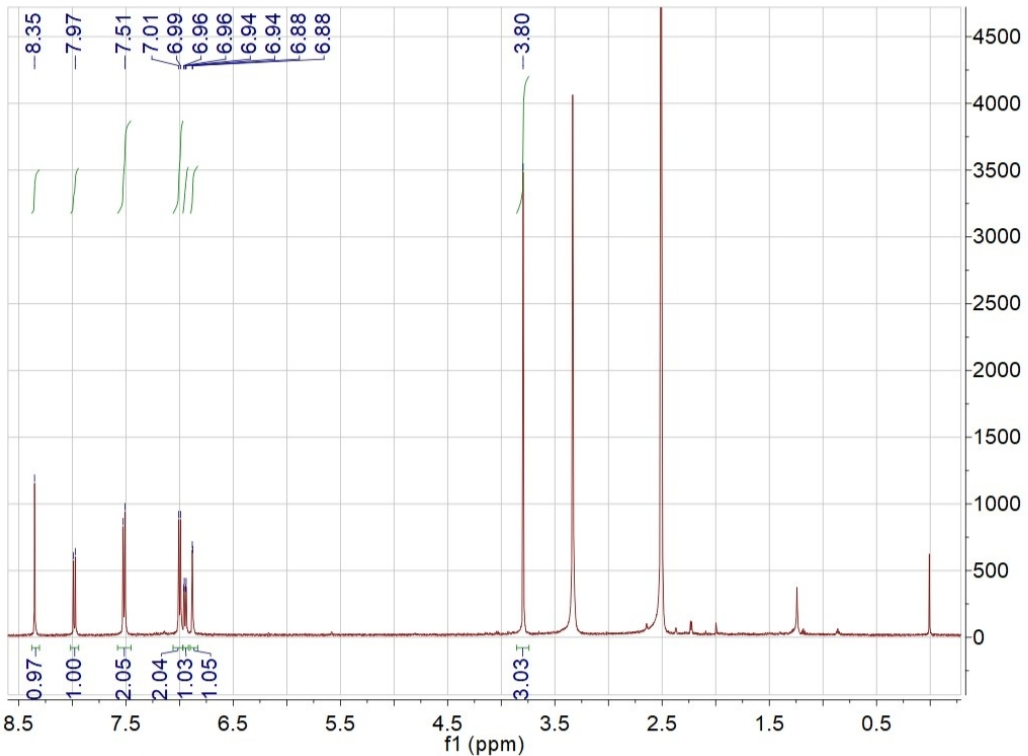


**Figure S12** 1HNMR analysis of compound 5.

1H NMR (400 MHz, in DMSO-d6) δ 3.8 (3H, s, 4'-OCH3), 6.88 (1H, d, 8-H), 6.94 (1H, dd, 6-H), 6.99 (2H, d, 3',5'-H), 7.51 (2H, d, 2',6'-H), 7.97 (1H, d, 5-H), 8.35 (1H, s, 2-H).


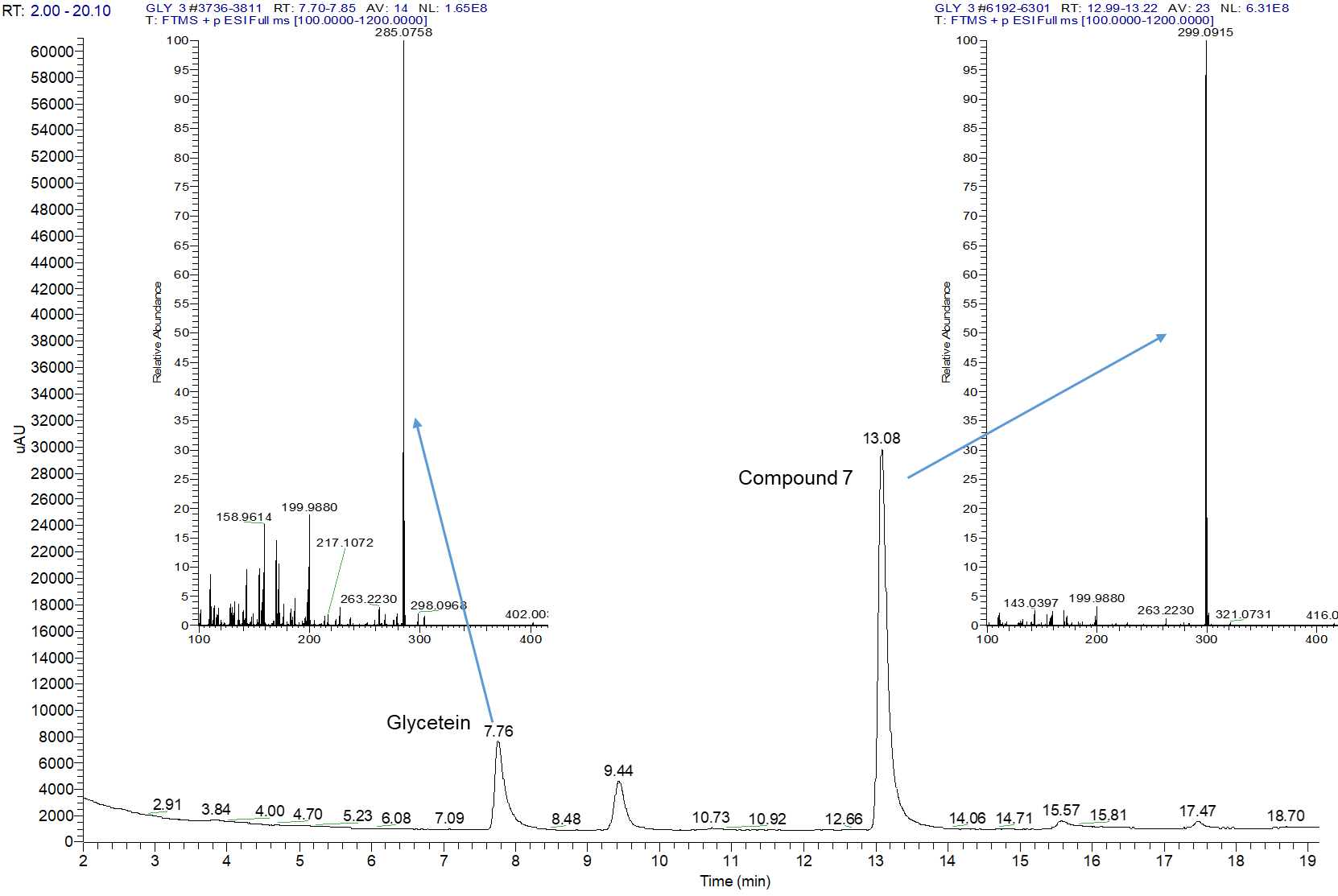


**Figure S13** Mass spectrometry analysis of the reaction product compound 7 catalyze by GmOMT3.


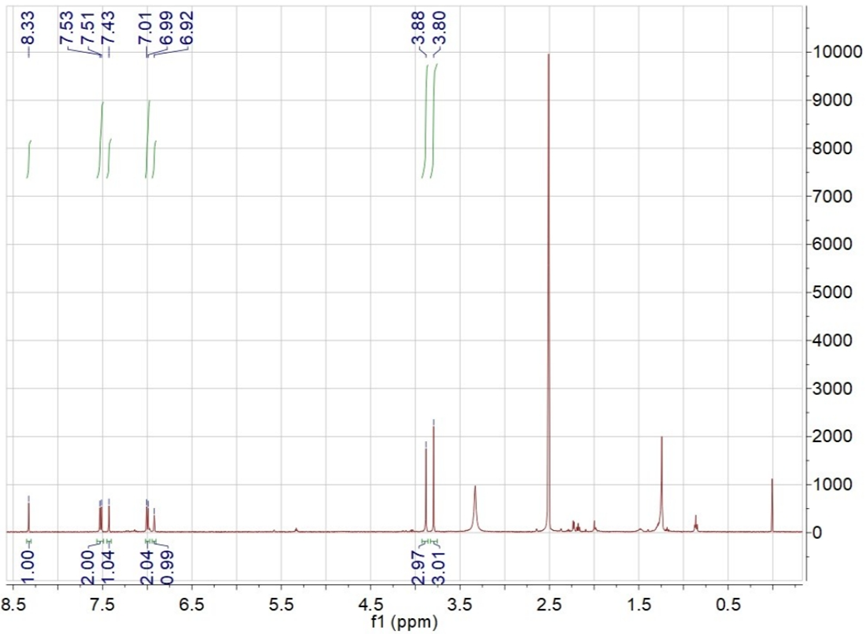


**Figure S14** 1HNMR analysis of compound 7.

1H NMR (400 MHz, in DMSO-d6) δ 3.8 (3H, s, 4'-OCH3), 3.88 (3H, s, 6-OCH3), 6.92 (1H, s, 8-H), 7 (2H, d, 3',5'-H), 7.43 (1H, s, 5-H), 7.52 (2H, d, 2',6'-H), 8.33 (1H, s, 2-H).


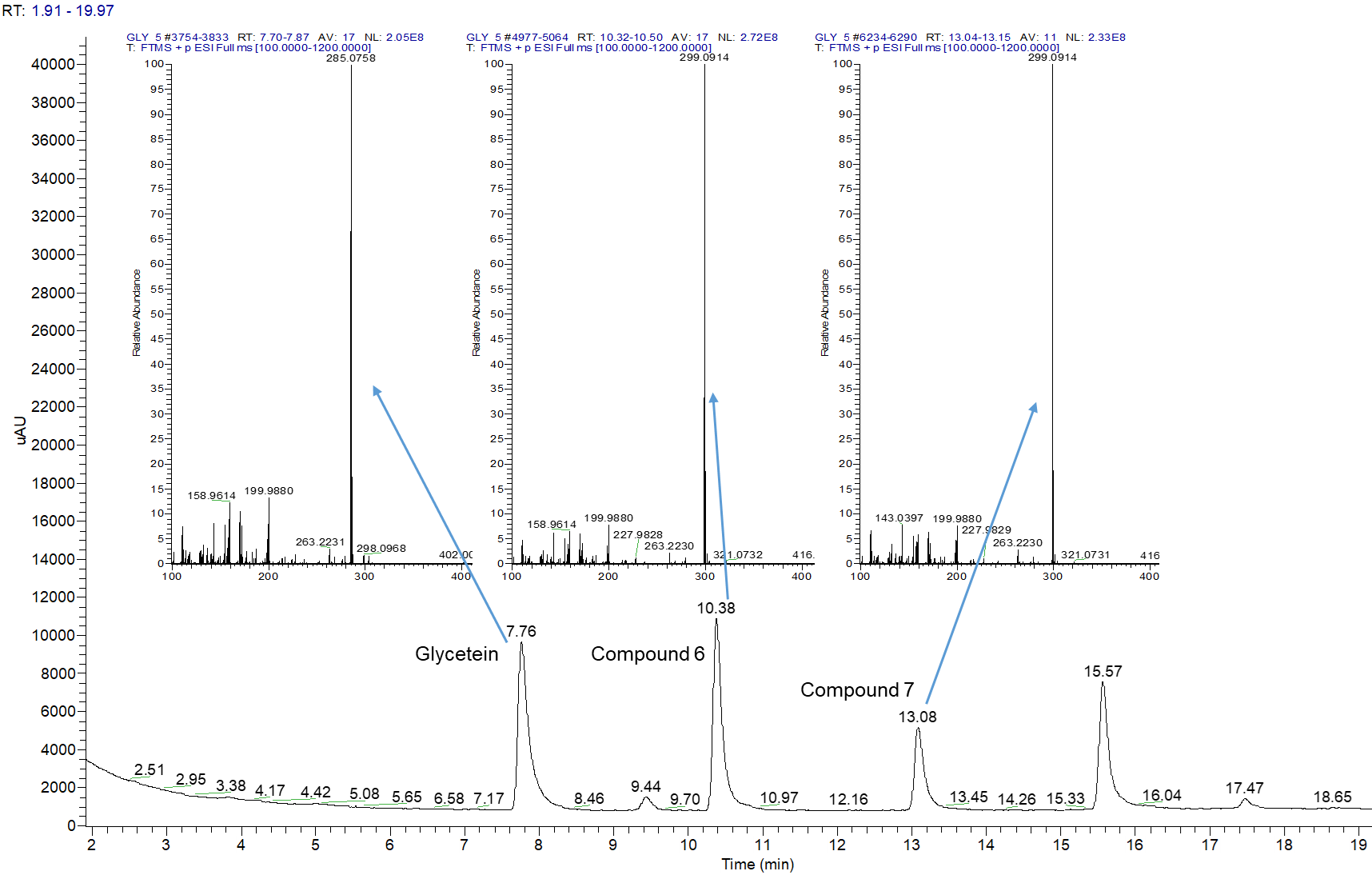


**Figure S15** Mass spectrometry analysis of the reaction product compound 6 and 7 catalyze by GmOMT5.


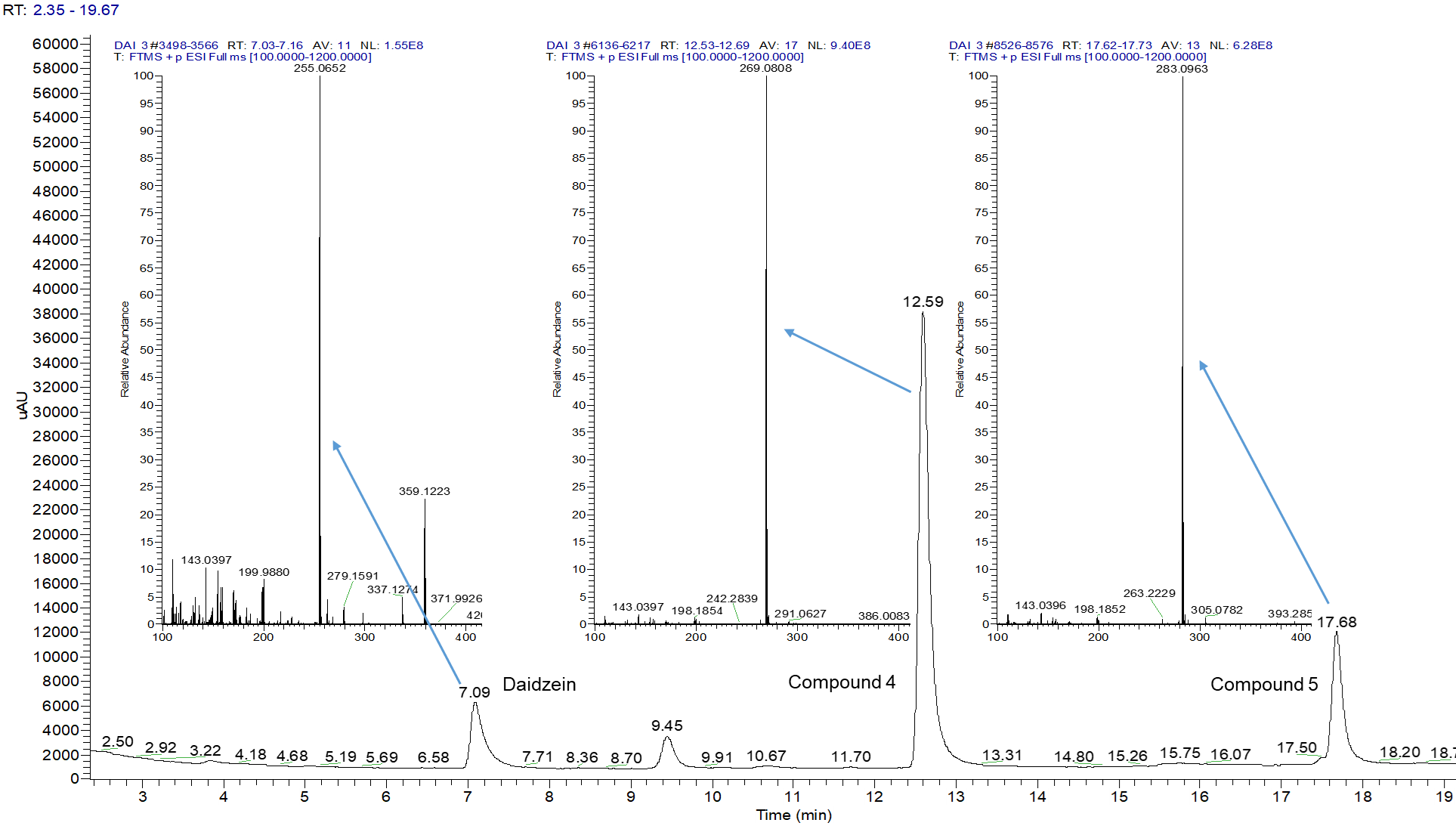


**Figure S16** Mass spectrometry analysis of the reaction product compound 4 and 5 catalyze by GmOMT5.
